# Eosinophils mediate SIgA production triggered by TLR2 and TLR4 to control *Ascaris suum* infection in mice

**DOI:** 10.1371/journal.ppat.1010067

**Published:** 2021-11-16

**Authors:** Denise Silva Nogueira, Luciana Maria de Oliveira, Chiara Cássia Oliveira Amorim, Ana Clara Gazzinelli-Guimarães, Fernando Sérgio Barbosa, Fabrício Marcus Silva Oliveira, Lucas Kraemer, Matheus Mattos, Mariana Santos Cardoso, Nathália Maria Resende, Marianna de Carvalho Clímaco, Deborah Aparecida Negrão-Corrêa, Ana Maria Caetano Faria, Marcelo Vidigal Caliari, Lilian Lacerda Bueno, Soraya Gaze, Remo Castro Russo, Pedro Henrique Gazzinelli-Guimarães, Ricardo Toshio Fujiwara

**Affiliations:** 1 Department of Parasitology, Institute of Biological Sciences, Universidade Federal de Minas Gerais, Belo Horizonte, Brazil; 2 Centro de Ciências Biológicas e da Saúde, Universidade Federal de Sergipe, Aracajú, Brazil; 3 Centro Universitário de Formiga, Formiga, Brazil; 4 Department of Morphology, Institute of Biological Sciences, Universidade Federal de Minas Gerais, Belo Horizonte, Brazil; 5 Department of Physical Education, Universidade Federal de Lavras, Lavras, Brazil; 6 Department of Biochemistry and Immunology, Institute of Biological Sciences, Universidade Federal de Minas Gerais, Belo Horizonte, Brazil; 7 Department of Pathology, Institute of Biological Sciences, Universidade Federal de Minas Gerais, Belo Horizonte, Brazil; 8 René Rachou Institute, Oswaldo Cruz Foundation–FIOCRUZ, Belo Horizonte, Brazil; 9 Department of Physiology and Biophysics, Institute of Biological Sciences, Universidade Federal de Minas Gerais, Belo Horizonte, Brazil; 10 National Institute of Allergy and Infection Diseases, National Institutes of Health, Bethesda, Maryland, United States of America; University of California Riverside, UNITED STATES

## Abstract

Human ascariasis is the most prevalent but neglected tropical disease in the world, affecting approximately 450 million people. The initial phase of *Ascaris* infection is marked by larval migration from the host’s organs, causing mechanical injuries followed by an intense local inflammatory response, which is characterized mainly by neutrophil and eosinophil infiltration, especially in the lungs. During the pulmonary phase, the lesions induced by larval migration and excessive immune responses contribute to tissue remodeling marked by fibrosis and lung dysfunction. In this study, we investigated the relationship between SIgA levels and eosinophils. We found that TLR2 and TLR4 signaling induces eosinophils and promotes SIgA production during *Ascaris suum* infection. Therefore, control of parasite burden during the pulmonary phase of ascariasis involves eosinophil influx and subsequent promotion of SIgA levels. In addition, we also demonstrate that eosinophils also participate in the process of tissue remodeling after lung injury caused by larval migration, contributing to pulmonary fibrosis and dysfunction in re-infected mice. In conclusion, we postulate that eosinophils play a central role in mediating host innate and humoral immune responses by controlling parasite burden, tissue inflammation, and remodeling during *Ascaris suum* infection. Furthermore, we suggest that the use of probiotics can induce eosinophilia and SIgA production and contribute to controlling parasite burden and morbidity of helminthic diseases with pulmonary cycles.

## Author summary

Ascariasis is the most prevalent but neglected tropical disease in the world, affecting more than 450 million people, mainly children. The parasites life cycle can be divided into the following two distinct phases after the initial infection: (i) the first stage called larval ascariasis involves migration of parasitic larval stages through several tissues (intestinal mucosa, blood circulation, liver, and lung/airways) and is marked by mechanical injuries, followed by an intense local inflammatory response, (ii) the second involves the establishment of adult worms in the lumen of the small intestine, causing the despoilment of nutrients and secretory/excretory parasitic products that downmodulate host immune response and characterize the chronic infection. Innate and adaptive immune responses are intrinsically connected and their cross-talk is very important for host homeostasis and is crucial in dealing with helminthiasis. In this study, we evaluated the importance of eosinophils mediating IgA production in mucosal sites, tissue inflammation, and remodeling, as well as controlling parasite burden during experimental larval ascariasis. This study provides a new perspective suggesting that stimulation by probiotics may prevent the lung pathology associated with ascariasis.

## Introduction

Lungs are important organs of homeostasis comprising a complex network that harbors the interaction between resident lung cells and leukocytes, are responsible for respiratory mucosa protection, and provide constant surveillance against potential pathogens. Pulmonary epithelial cells, resident macrophages, dendritic cells, and myeloid cells of the airway mucosa trigger an immune response against invading pathogens using a series of pattern recognition receptors (PRRs). Toll-like Receptors (TLRs) are PPRs, the activation of which releases a wide range of immune mediators such as complement proteins, antimicrobial peptides, antibodies, cytokines, and chemokines, to control infection. These mediators orchestrate a series of actions against pathogens, coordinating leukocyte influx and activation at the site of infection, consequently inducing adaptive immunity, which leads to long-term immunity [1].

Ascariasis is the most prevalent helminth infection, affecting approximately 450 million people worldwide [2–4]. The relationship between the components of innate and adaptive immunity in ascariasis remains unclear. However, eosinophils, the most prominent cell type observed in helminthiasis show a variety of immune-regulatory and pro-inflammatory roles ranging from antigen presentation to the release of preformed cytokines, chemokines, lipid mediators, and cytotoxic granule proteins in ascariasis [5–7]. Moreover, multiple growth factors and cytokines expressed by eosinophils have been implicated in the promotion of tissue remodeling and fibrosis in airway diseases [7]. Several studies have demonstrated that eosinophils are associated with resistance to helminths, either in conjunction with humoral immune components, such as antibodies or complements or with innate cells. Furthermore, several studies have suggested the importance of eosinophils in the control of *Ascaris suum* after single- and re-infection [8,9].

Helminth infections with lung worm cycle/passage induce pulmonary inflammation, generating a T-helper (Th) type 2 immune response, similar to that seen in allergic airway diseases, such as asthma. This pathology caused by ascariasis, characterized by eosinophilia, pulmonary histological changes, goblet cell hyperplasia, and increased mucus production, leads to Loeffler’s syndrome [3,10,11]. Ascariasis has recently been associated with the onset of allergic airway diseases, especially because of the Th2 polarized response following primary infection [12,13] and further exacerbation by a mixed Th2/Th17 response following multiple exposures [9].

Migrating *Ascaris* larvae induce a robust local response in the lungs of experimentally infected mice. The likely source of interleukin (IL)-5, although not yet elucidated in ascariasis, appears to be type 2 innate lymphoid cells (ILC2s), as has been demonstrated in murine models of allergic asthma and other helminth infections [11,14–16]. ILC2s respond to IL-25 and IL-33 cytokines derived mainly from epithelial cells in response to damage, and in turn, produce type 2 cytokines, such as IL-5, IL-9, and IL-13, and secrete IgE, IgG1, and IgA [16]. Eight days post-infection, at the peak of *Ascaris* larvae migration in the lungs is characterized by marked IL-6 production that may be related to a prominent neutrophil infiltration that occurs concomitantly. When the larvae start to leave the lungs and migrate to the small intestine, the neutrophil infiltrate in the lungs is replaced by both mononuclear cells and eosinophils [8]. Eosinophils are also implicated in the regulation and maintenance of alternatively activated macrophages, which further confirms the role of eosinophils as a critical regulator of type 2 responses [16].

Although eosinophils are considered important in anthelmintic immune responses [6,17], studies on eosinophil homeostatic functions have also demonstrated their participation in plasma cell survival and regulation of secretory IgA (SIgA) production in the intestinal mucosa [5,18–20]. Studies on the parasite *Teladorsagia circumcincta* demonstrated that SIgA and eosinophil kinetics were similar and that the interaction between the two components of the immune response contributes to parasite size reduction [17]. Similarly, in experimental infections of *Cooperia oncophora*, eosinophils and SIgA were correlated with reduced parasite burden [21].

The effect of eosinophil absence on SIgA production has also been demonstrated using a GATA1^-/-^ mouse model [18,19] wherein eosinophil absence leads to a reduction in antibody production in the intestinal mucosa. Therefore, in this study, we explored the role of eosinophils during larval ascariasis and investigated the relevance of eosinophils in SIgA production, parasite burden control, and tissue repair. Furthermore, we highlight the importance of TLR2 and TLR4 receptor signaling in eosinophil recruitment and SIgA production, resulting in effective cellular and humoral immune responses against larval ascariasis in mice.

## Materials and methods

### Ethics statement

All procedures in this study were approved by the Ethics Committee for Animal Experimentation (CETEA) of Federal University of Minas Gerais (UFMG), Brazil (Protocol # 04/2012, Protocol # 054/2012, and Protocol # 187/2014). The animals were maintained in accordance with the recommendations of the Brazilian College of Animal Experimentation guidelines (COBEA).

### Animals

Wild type (WT) BALB/c and WT C57BL/6 mice were obtained from the Central Animal Facility of the Federal University of Minas Gerais, Brazil. GATA1^-/-^ mice were provided by the Laboratory of Immunopharmacology, Institute of Biological Sciences, Federal University of Minas Gerais, Belo Horizonte, Minas Gerais, Brazil. TLR2^-/-^ and TLR4^-/-^ mice were provided by the Laboratory of Cellular and Molecular Immunology, René Rachou Institute, Oswaldo Cruz Foundation-FIOCRUZ, Belo Horizonte, Minas Gerais, Brazil.

### Experimental design

The following four independent experimental designs were used in this study depending on the objective: 1) To evaluate the kinetics of the immunological and parasitological parameters, WT C57BL/6 and BALB/c mice were divided into seven groups—a control group = non-infected (NI) and six single-infected groups (SI). 2) To evaluate the role of eosinophils in TLR-deficient mice, immunological and parasitological parameters were observed at 8 dpi. The animals were divided into two groups—a control group = non-infected (NI) and a single-infected group (SI). 3) To evaluate the effect of probiotics on immunological and parasitological parameters, animals were divided into four groups—a non-infected group treated with placebo (NI-M17), a non-infected group treated with probiotics (NI-NCDO), a single-infected group treated with placebo (SI-M17), and a single-infected group treated with probiotic (SI-NCDO). 4) To evaluate the role of eosinophils in single and multiple infections, GATA1^-/-^ and WT BALB/c mice were divided into three groups—a non-infected group (NI), single-infected group (SI), and re-infected group (RE). Mice from the NI groups were administered phosphate buffered saline (PBS) only. Mice from the SI group were treated with two doses of PBS followed by one dose of suspension containing 2500 fully embryonated eggs of *A*. *suum* (single-infection), maintaining an interval of 14 days between doses. Mice from the RE group were administered three doses of a suspension containing 2500 fully embryonated eggs of *A*. *suum*, maintaining an interval of 14 days between doses (re-infection). 5) To evaluate the participation of eosinophils in probiotic-mediated SIgA modulation, GATA1^-/-^ and WT BALB/c mice were divided into four groups—a treated with placebo and non-infected (PBS NI), treated with placebo and infected with *A*. *suum* (PBS SI), treated with NCDO (2118) and non-infected, and, treated with NCDO (2118) and infected with *A*. *suum* (NCDO SI). Tissue harvesting for parasitological and immunological evaluation was performed as previously described [9] at all time points of kinetics experiments or following the peak migration of larvae in the liver, lungs, and intestine, which were observed on days 4, 8, and 12 of infection, respectively [8]. The experimental design and results are outlined in the figures.

### Bacterial growth conditions for probiotic diet

A gram-positive and non-invasive bacterial strain from *Lactococcus lactis* subsp. *lactis* (NCDO 2118) was used to assess the impact of a probiotic diet on ascariasis. Bacteria were grown as previously described [22], with some modifications. First, for experimental design 3, bacteria were cultured for 18 to 21 hours, at 30°C in M-17 broth medium (Sigma) supplemented with 0.5% glucose and 10 μg/mL of chloramphenicol (Sigma) without agitation. Each animal was given a volume of 5 mL containing either M17 medium (group M17) or freshly cultured bacteria in the stationary growth phase (NCDO group) daily. An estimated dose of 1 × 10^9^ CFU/mL bacteria per day for 22 days was ingested by each mouse [22].

For experimental design 5, the bacterial culture was prepared similarly, but without the use of antibiotics. Upon reaching the stationary phase, the culture was centrifuged at 4000 g at 4°C for 10 minutes. The supernatant was discarded, and the pellet was resuspended in PBS. This solution was administered in mice intragastrically, with the aid of a gavage needle, at a concentration of 1 × 10^9^ CFU/100μL bacteria per day for 22 days [23].

During the experimental period, the mice had unlimited access to food and water.

### Parasites

Adult *A*. *suum* worms were harvested from pigs from a Brazilian slaughterhouse (Belo Horizonte, Minas Gerais, Brazil). Eggs were isolated from the uteri of female worms by gentle mechanical maceration and purified using cell strainers (70 μm). The isolated eggs were incubated with 0.2 M H_2_SO_4_ for embryonation, as described by Boes et al. [24]. After the 100^th^ day of culture, which corresponds to the peak of larval infectivity [8], fully embryonated eggs were used in experimental infections and some were allowed to hatch for harvesting larvae to obtain antigens.

### Experimental infection and parasitological analysis

The mice were administered either 200 μL of PBS or 2500 embryonated eggs suspended in 200 μL PBS using a gavage needle, according to the group, time point, and experimental design. To assess the parasite burden, tissues were collected, sliced using scissors, and placed in a Baermann apparatus for 4 hours in PBS at 37°C. The recovered larvae were then fixed with 1% formaldehyde in PBS and counted under an optical microscope. Total parasite burden in the kinetic experiment was assessed as the total number of larvae recovered from the liver, lungs, and intestines at all six time points. In all other experimental designs, the parasite burden was assessed as the number of larvae recovered from the liver, lungs (parenchyma + airways), or intestine, separately.

### Attainment and analysis of bronchoalveolar lavage (BAL)

Bronchoalveolar lavage (BAL) was obtained and used for the analyses of cellularity, blood loss, protein leakage, SIgA levels in the BAL, and the number of larvae in the airways. For obtaining BAL, the mice were administered a lethal dose of anesthetic (xylazine 45 mg/kg and ketamine 120 mg/kg). The animals were then tracheostomized and a 1.7 mm catheter was inserted into the trachea to obtain the BAL through two infusions and aspirations using 1 mL of PBS. The BAL obtained was filtered through 40 μm cell filters (BD, USA) to recover *A*. *suum* larvae, which were used to assess the parasite burden in the airways [9]. Filtered BAL was centrifuged at 3000 g for 10 minutes and the pellet was used to determine the total number of leukocytes and their subpopulations (macrophages, lymphocytes, eosinophils, and neutrophils) using optical microscopy. The supernatant was used to quantify blood loss, protein leakage, and SIgA levels by ELISA.

### Assessment of respiratory mechanics

For assessing respiratory mechanics, mice were anesthetized using subcutaneous ketamine and xylazine injection (8.5 mg/kg xylazine and 130 mg/kg ketamine) to maintain spontaneous breathing under anesthesia, following which they were tracheostomized, placed in a body plethysmograph, and connected to a computer-controlled ventilator (Forced Pulmonary Maneuver System, Buxco Research Systems, Wilmington, North Carolina USA), as previously described by Nogueira and colleagues (2016) [9]. An average breathing frequency of 160 breaths/min was imposed on the anesthetized animal by pressure-controlled ventilation until a regular breathing pattern and complete expiration at each breathing cycle was obtained. Under mechanical respiration, the dynamic compliance (Cdyn) and lung resistance (Rl) were determined using resistance and compliance RC tests. To measure the forced vital capacity (FVC), a quasi-static pressure-volume maneuver was performed, allowing lung inflation to a standard pressure of +30 cm H_2_O and then slowly exhalation until a negative pressure of -30 cm H_2_O. Quasi-static chord compliance (from 0- to +10 cm H_2_O) was calculated with this maneuver considering the volume/pressure of the expiration. With the fast flow volume maneuver, the lungs were first inflated to +30 cm H_2_O and immediately connected to a highly negative pressure to enforce expiration until -30 cm H_2_O. Forced expiratory volume (forced expiratory volume at 100 milliseconds, FEV100) was recorded during this maneuver. Suboptimal maneuvers were rejected, and for each test in every mouse, at least three acceptable maneuvers were conducted to obtain a reliable mean for all numeric parameters.

### Histopathological analysis

For histopathological analysis after single-infection, mice were euthanized and the left lobe of the lung was dissected, fixed in 10% buffered formalin (pH 7.2), and embedded in paraffin. Tissue sections (5 μm thick) were mounted on glass slides and stained with hematoxylin and eosin (H&E) for inflammation analysis, Masson’s trichrome or Picrosirius Red for quantification of collagen neoformation as well as determination of the type of collagen fiber under polarized light illumination. The tissues were then imaged using a standard light microscope (Leica DM5000). For studying the morphometric parameters of inflammation, the images were analyzed from 15 randomly selected fields (total area 1.12 × 10^6^ μm^2^) on a single slide per animal. The segmentation allowed us to select the nuclei of all cell types present in the lung and to exclude the cell cytoplasm and other structures present in the histological sections. Using this process, a binary image was created, and the nuclei of all cell types present in the lung were counted. The difference between the number of cell nuclei present in infected mice and that in non-infected animals was considered as the number of inflammatory cells. For studying the morphometric of collagen neoformation, the images were analyzed from 20 randomly selected fields (total area 1.5 × 10^6^ μm^2^) on a single slide per animal. Collagen neoformation in the lung was quantified by measuring the collagenized areas present in the sections, and the difference between the collagenized areas in the infected and non-infected mice was considered as fibrosis process. All sections were viewed using a 40X objective and the images were digitized using a Leica DFC340FX microcamera associated with Leica DM5000B microscopy and analyzed using the image processing and analysis software Leica Qwin V3 (Leica Microsystems, Wetzlar, Germany).

For histopathological analyses after re-infection experiments, mice were euthanized on the 8^th^ day after infection, and their lungs were removed. The left lobe was fixed in a 10% solution of formaldehyde (Synth, Brazil) in PBS for 72 hours. After processing in alcohol and xylol, tissue fragments were embedded in paraffin and 4 μm thick sections were obtained and stained with H&E for morphometric quantification of inflammatory and Gomori’s trichrome for morphometric quantification of collagen deposition and fibrosis. Images from the tissue sections were collected using a 20X objective of the Axiolab-Carl Zeiss microscope (Oberkochen, Germany) and a Samsung SDC-415 micro-camera (Seoul, South Korea). Forty random images were captured with a 20X magnification lens, covering an area of 3.2 × 10^6^ μm^2^ of lung analyzed. The KS400 software was used to select all pixels of the lung tissue in the real image for creating a binary image, digital processing, and calculation of the area in μm^2^ of the interalveolar septum and fibrosis [25].

### Tissue IgA immunostaining

To further evaluate the expression of lung IgA, we performed immunohistochemistry as previously described [26]. Briefly, tissue sections were deparaffinized, hydrated, and washed in PBS (pH 7.2). Endogenous peroxidase activity was eliminated by treatment with H_2_O_2_ 40 v/v solution in 0.2% PBS and non-specific binding was blocked by incubation with goat serum diluted at 1:40. The sections were then incubated with polyclonal anti-IgA (Southern Biotechnology Associates, Birmingham, USA) diluted at 1:60 for 18 hours, and then incubated with biotinylated rabbit anti-goat IgG at 1:150 (Bethyl Laboratories Inc., Montgomery, USA). The reaction was then revealed using DAB 0.05% in H_2_O_2_ 40% v/v in 0.2% PBS. PBS-treated sections were used as negative controls (S1 Fig). All sections were stained with H&E, desihydrated, and diaphanized for entellan (Meck Millipore) assembly.

### Adult worm and infective larvae antigen production

*A*. *suum* adult worm and larval antigen production was performed as described previously [27]. Briefly, larvae antigen was used to induce the hatching of fully embryonated *A*. *suum* eggs, a standard protocol for *Toxocara canis* was used with some modifications [28]. Embryonated *A*. *suum* eggs were incubated in RPMI-1640 medium (SIGMA, USA; pH 7.2) supplemented with 4% penicillin/streptomycin (Invitrogen, USA), and placed in 24-well plates for larval hatching. To produce L3 larvae antigen, purified larvae were collected and transferred to a 50 mL graduated tube, centrifuged at 800 g for 10 minutes at room temperature. The supernatant was discarded and the pellet was resuspended in 5 mL of PBS, and then sonicated (Cole Parmer Ultrasonic Homogenizer Power Supply 4710 Series, USA) at 60 W for 1 minute, with a 30-second interval for each cycle, totaling 10 cycles. After sonication, the extract was centrifuged at 800 g for 15 minutes at 4°C. The supernatant was then collected and stored at -80°C until use. The amount of protein in all antigenic extracts was measured using a commercial BCA protein assay kit (Thermo Fisher Scientific, USA), according to the manufacturer’s instructions.

Adult worm antigens were initially obtained by mechanical maceration of the parasites in PBS and then using a sonicator (Cole Parmer Ultrasonic Homogenizer Power Supply 4710 Series, USA). The macerated extract was kept under ice-cooling and then sonicated at 60 W for 1 minute, with a 30-second interval for each cycle, totaling five cycles. Finally, the protocol was performed as previously described for larval antigens.

### Cytokine profile

To determine the cytokine profile in the lungs, the lungs were homogenized using a tissue homogenizer (Power Gen 125 –Fisher Scientific Pennsylvania, USA) in 100 mL of PBS supplemented with protease inhibitors (0.1 mM phenylmethylsulfonyl fluoride (PMSF), 0.1 mM benzethonium chloride, 10 mM EDTA and 20 KI aprotinin A) and 0.05% Tween 20. The lung homogenates were then centrifuged at 8000 g for 10 minutes at 4°C and the supernatant was used to determine cytokine production. Production of IL-2, IL-4, IL-6, IL-10, IL-17A, IFN-γ, and TNF-α was assessed by flow cytometry (Th1/Th2/Th17 Cytometric Bead Array, BD Biosciences, USA) using a FACScan (BD Biosciences, USA) according to the manufacturer’s recommendations. IL-5, TGF-β, and IL-13 levels were measured using a sandwich ELISA kit (R&D Systems, USA) according to the manufacturer’s instructions. Finally, the intensity of the reaction was determined at a wavelength of 492 nm using an automated reader (VersaMax Tunable Microplate Reader, Molecular Devices), and the cytokine concentration (pg/mL) for each sample was calculated by interpolation using a standard curve fitted using the five- parameter logistic (5-PL).

### Eosinophil peroxidase and neutrophil myeloperoxidase assays

Eosinophil peroxidase (EPO) and neutrophil myeloperoxidase (MPO) activity in lung homogenates were measured according to the method described by Strath and modified by Silveira [29,30]. The tissues were homogenized (Power Gen 125-Fisher Scientific Pennsylvania, USA), the homogenate was centrifuged at 8000 g for 10 minutes at 4°C, and the pellet obtained was used to determine EPO and MPO activity. For the EPO assay, the pellet was homogenized in 950 μL PBS and 0.5% hexadecyltrimethylammonium bromide (Sigma Chemical Co, St. Louis, MO, USA), and then frozen-thawed three times using liquid nitrogen. The lysate was then centrifuged (1500 g, 4°C, 10 minutes) and the supernatant was distributed in a 96-well microplate (75 μL/well) (Corning, USA), followed by the addition of substrate (1.5 mM OPD and 6.6 mM H_2_O_2_ in 0.05 M Tris-HCl, pH 8.0) (75 μL/well), and incubation for 30 minutes at room temperature. The reaction was stopped by the addition of 50 μL of 1 M H_2_SO_4_ and absorbance was determined at 492 nm.

For the MPO assay, the pellet obtained after centrifugation was homogenized in 200 μL of buffer 1 solution (0.1 M NaCl, 0.02 M Na_3_PO_4_, 0.015 M Na_2_EDTA, pH 4.7), then centrifuged (1500 g, 4°C, 10 minutes). Then, 800 μL of buffer 2 solution (0.05 M NaPO_4_, 0.5% hexadecyltrimethylammonium bromide) was added to the pellet and this mixture was homogenized and then frozen-thawed three times using liquid nitrogen. The lysate was centrifuged (1500 g, 4°C, 10 minutes) and the supernatant was used for the enzymatic assay. To each well of a 96-well microplate, 25 μL of supernatant was distributed (Corning, USA) followed by the addition of 25 μL of substrate TMB (3.3’-5.5—tetramethylbenzidine + 1.6 mM dimethylsulfoxide), and 100 μL of 0.5 M H_2_O_2_ and incubated for 5 minutes at room temperature. The reaction was stopped by adding 100 μL of sulfuric acid (1 M H_2_SO_4_). Finally, the intensity of the reaction was determined at a wavelength of 450 nm using an automated reader (VersaMax Tunable Microplate Reader, Molecular Devices).

### Immunoenzymatic assays for evaluation of immunoglobulins

To determine Total SIgA levels, polystyrene microplates (NUNC, Roskilde, Denmark) were coated with 0.5 μg/mL Goat Anti-Mouse Ig, Human ads-UNLB (catalog no. 1040–01, Southern Biotechnology Associate Inc., USA). To evaluate *Ascaris*-specific immunoglobulin levels, the microplates were coated with 1 μg/mL of excretory/secretory (ES) products or adult worm antigens and incubated overnight at 4°C. Subsequently, they were washed with PBS containing 0.05% Tween 20 (Sigma Chemical Co. USA). Blocking was performed at room temperature for 2 hours with PBS + 3% BSA (Fitzgerald Industries, USA). Samples (BAL, intestinal lavage, or serum) were then added to the plates and incubated for at least 24 hours at 4°C. The plates were then washed 5 times using washing buffer (PBS-0,05% Tween20), and incubated at 37°C for 1 hour with biotinylated anti-IgA (1:500 for specific levels; catalog no. 1040–05), or (1:10000 for total levels: catalog no. 1040–08), or IgG1-3 (1:1000; catalog no. 1070–05, 1080–05, 1090–05, and 1100–05, respectively) and HRP-conjugated IgE (1:500; catalog no. 1110–05) (Southern Biotechnology Associate, Inc.), or HRP-conjugated IgM (1:1000; catalog no. 550588) (BD Pharmingen, Inc.). The plates were then washed and for distinguishing total and *Ascaris*-specific IgA, an additional incubation was performed using an HRP-streptavidin-conjugated solution for 2 hours (Horseradish Peroxidase) (Streptavidin-HRP, R&D Systems, USA). The plates were then incubated with substrate O-phenylenediamine (OPD, SIGMA Chemical Co., + 30% H_2_O_2_). Finally, the signal intensity was determined at a wavelength of 492 nm using an automated reader (VersaMax Tunable Microplate Reader, Molecular Devices).

For quality control, positive and negative controls were included on all plates. Serial dilutions of IgA standards (Mouse IgA standard, Southern Biotechnology) were used in all complete total SIgA evaluation plates. Concentrations of total SIgA (μg/mL) were determined by interpolation of the sample optical density (OD) in a standard curve fitted using the five-parameter logistic (5-PL). Levels of *Ascaris*-specific Igs are expressed as OD.

### Statistical analyses

Statistical analyses were performed using the GraphPad Prism 8 software (GraphPad Inc., USA). The Grubb’s test was used to detect possible outliers in the groups. The Mann-Whitney test was used for comparing parasite burden and the areas of lesions in the liver and lungs. One-way ANOVA followed by Tukey´s multiple comparisons test was used to evaluate differences between groups in kinetics experiments. Pearson’s Linear Regression was used to evaluate the correlation. Data from EPO and MPO assays and pulmonary mechanics, hemoglobin, protein levels, and BAL cellularity were analyzed using the Kruskal-Wallis test followed by Dunn’s test. Finally, a two-way ANOVA with multiple comparisons was performed to assess differences between the groups as a function of time. All values were considered significant when at p ≤ 0.05.

## Results

### Mucosal SIgA production correlates with eosinophilia and controls parasite burden during larval ascariasis in mice

**Fig 1:**
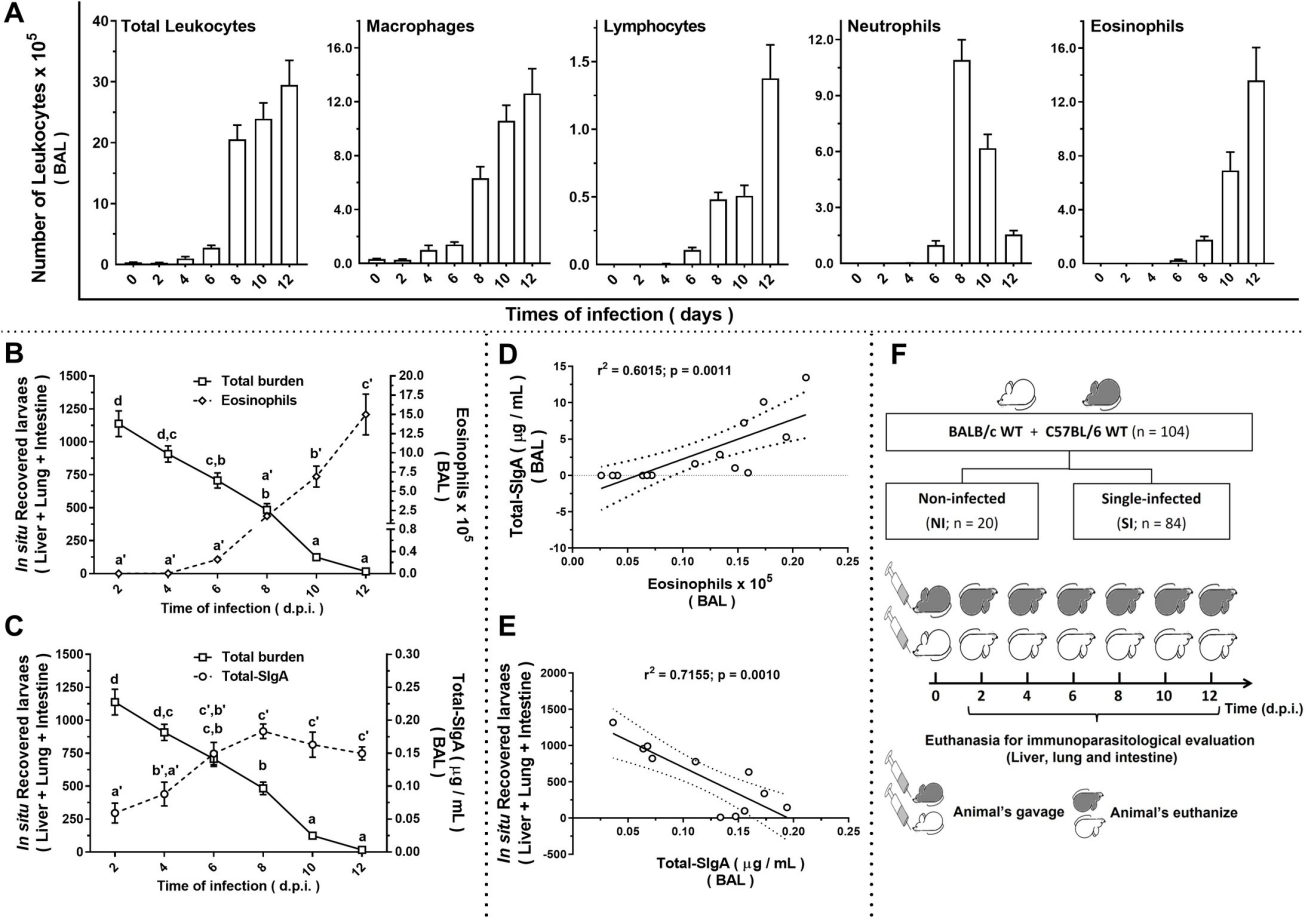
Kinetics of immune response and parasite burden during experimental ascariasis in mice. Number of leukocytes and their cell subpopulations in bronchoalveolar lavage fluid (BAL) (n = 7) (A). Coincidence curve showing inverse relationship observed between total parasite burden and the number of eosinophils (n = 7) (B) and total SIgA levels (n = 7) (C) in BAL. Scatterplot graphics showing a positive correlation between total SIgA levels and the number of eosinophils from BAL (n = 7) (D) and a negative correlation between total parasite burden and total SIgA levels in BAL (n = 7) (E) on the8^th^ dpi. Schematic representation of experimental design (F). One-way ANOVA followed by Tukey´s multiple comparisons test was used to evaluate differences between groups in (A), and Pearson’s Linear Regression was used to evaluate the correlation in (D) and (E). The letters in (B) and (C) indicate statistically significant difference between mean values, where [a < b < c < d] and [a’ < b’ < c’]. Significant differences between groups (p < 0.05) are represented by the p values in A, D, and E graphs. Data are presented as mean ± SEM.

The relationship between eosinophils and SIgA in *A*. *suum* infection model was initially evaluated by comparing immune response at different timeframes during larval migration kinetics in WT BALB/c and WT C57BL/6 mice. Immune responses associated with larval migration were assessed based on leukocyte influx in the lungs and SIgA production. After *A*. *suum* infection, there was a progressive increase in the total number of leukocytes, macrophages, lymphocytes, and eosinophils from the 8^th^ dpi to 12^th^ dpi as larvae migrate through the lungs. Whereas the number of neutrophils increased on the 8^th^ dpi but decreased on the 10^th^ and 12^th^ dpi (Fig 1A and S1 Table). Interestingly, we found similar kinetics of eosinophil influx (Fig 1B) and total SIgA levels (Fig 1C) in the lung mucosa, with a progressive increase after the 6^th^ dpi. This correlation suggests that eosinophils contribute to control of the parasite burden and is relevant to SIgA production during larval ascariasis in mice. In mouse airways, increased SIgA levels on the 8^th^ dpi was positively correlated with eosinophil influx (Fig 1D) and negatively correlated with the number of larvae (Fig 1E). Fig 1F shows the experimental design. WT C57BL/6 and WT BALB/c mice were divided into non-infected (n = 20) and single-infected (n = 84) groups. The data in Fig 1 represent the mean of the results of the two strains, as there were no significant differences in the responses observed between the two strains.

Apart from our objective, we also verified that there was a significant increase in the production of IgM in the BAL and serum of WT C57BL/6 mice on the 10^th^ dpi. However, there was no significant difference between WT BALB/c and WT C57BL/6 strains (S2 Fig).

### TLR2 and TLR4 mediates mucosal SIgA production and eosinophilia that controls parasite burden during larval ascariasis in mice

**Fig 2:**
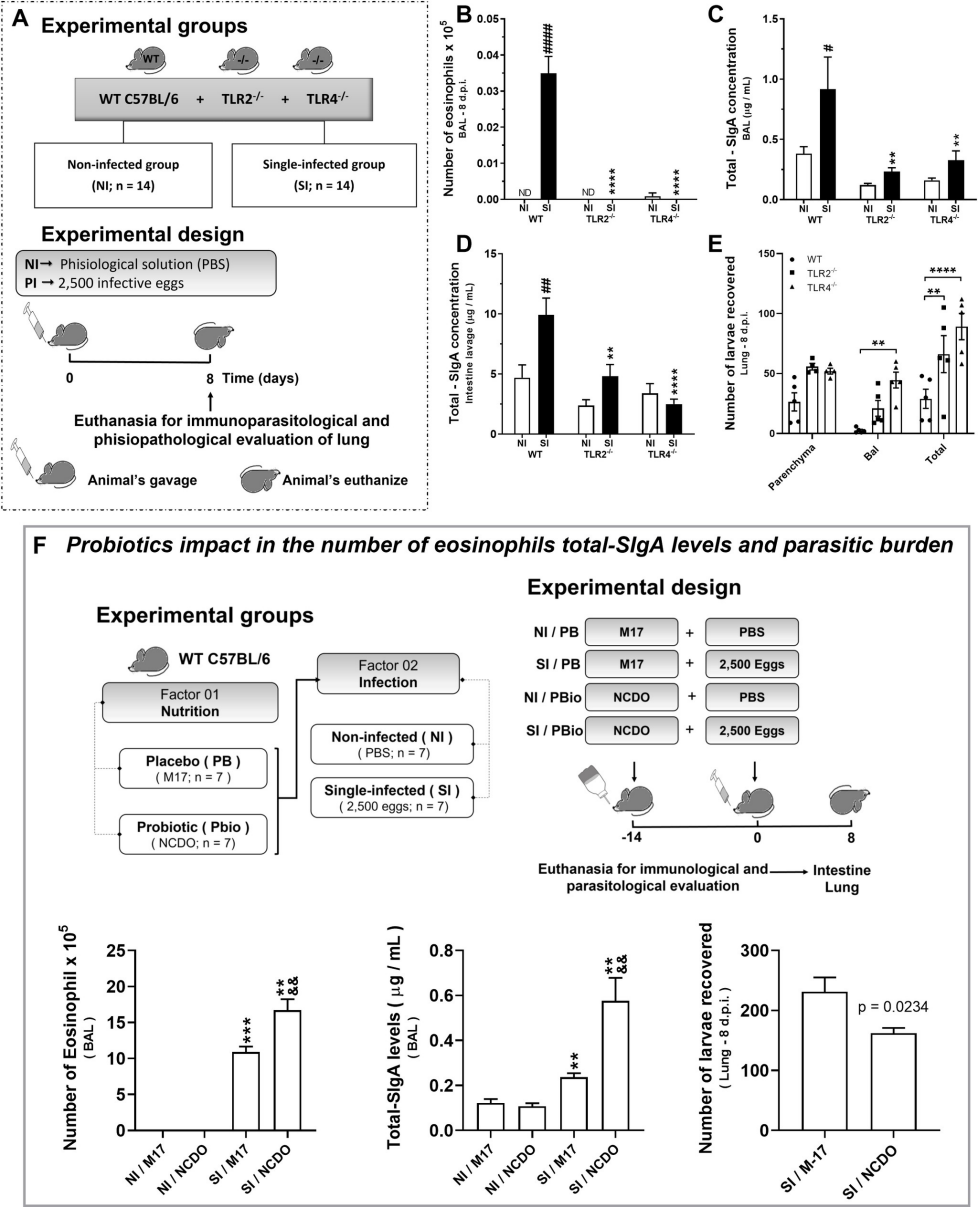
TLR2 and TLR4 receptors trigger eosinophilia and mucosal SIgA production that control larval ascariasis. Schematic representation of the experimental design using TLR2^-/-^, TLR4^-/-^ and WT C57BL/6 mice (A). Number of eosinophils recovered from BAL (n = 14) (B), total SIgA levels from BAL (n = 14) (C) and intestine lavage (n = 14) (D) on the 8^th^ dpi. Total parasite burden, that is, the number of larvae recovered in lung parenchyma and airways of WT C57BL/6, TLR2^-/-^ and TLR4^-/-^ mice on the 8^th^ dpi (n = 14) (E). Effect of probiotic *Lactococcus lactis* (NCDO 2118) on immunological and parasitological parameters during the acute phase of experimental ascariasis in mice, namely, number of eosinophils in BAL (n = 7), total SIgA (n = 7), and coincident reduction in parasite burden (n = 7) (F). Two-way ANOVA followed by Sidak´s multiple comparisons test was used to evaluate differences between groups (B-E) and the Mann-Whitney test was used to assess differences between the groups (F). Differences were considered significant at p ≤ 0.05 and are represented by symbols in the graphs. * represents differences between knockout and WT C57BL/6 mice groups and # represents differences between non-infected and single-infected groups from same strain (B, C, and D); and, the * represents differences between the single-infected and non-infected group and & represents differences between the NCDO and M17 groups (F). [1 symbol = p < 0.05], [2 symbols = p < 0.01], [3 symbols = p < 0.001]. Data are presented as mean ± SEM.

After demonstrating the relationship between SIgA levels and eosinophils controlling larval migration, and after verifying the increase in the expression of TLR2 and TLR4 in single-infected mice (S3 Fig), we assessed the potential role of TLRs in triggering SIgA production and eosinophilia. Thus, we investigated the immune response and parasite burden during *A*. *suum* infection in mice deficient for Toll-like receptor 2 or Toll-like receptor 4 (TLR2^-/-^ or TLR4^-/-^) (Fig 2A). We found that both TLR2 and TLR4 receptors are required to induce a protective immune response, as a significant reduction in eosinophil numbers in the airways was observed in TLR2^-/-^ and TLR4^-/-^ mice (Fig 2B) compared to WT mice. Furthermore, we also found lower levels of total SIgA in BAL and intestine lavage from TLR2^-/-^ and TLR4^-/-^ deficient mice, compared to that of WT mice (Fig 2C and 2D). This reduction in immune response was due to a significant increase in the number of *Ascaris* larvae recovered from lung tissue and airways from TLR2^-/-^ and TLR4^-/-^ mice. The most marked burden occurred in the airways of TLR4^-/-^ mice (Fig 2E), suggesting that TLR deficiency also affects the number of larvae with the potential to complete their life cycle in the intestine.

Considering the relevant role of TLRs in *A*. *suum* infection, we investigated the use of probiotics in inducing a response mediated by TLR receptors in the course of infection. The bacterium *Lactococcus lactis* (NCDO 2118) is a gram-positive bacterium that has lipoteichoic acid (LTA) on its surface. These PRRs are conserved molecular structures that can be recognized by TLR2 [31]. We found an increase in SIgA production and the number of eosinophils, as well as a concomitant reduction in parasite burden at pulmonary sites in mice treated with the probiotic bacteria during infection with *A*. *suum* when compared to mice that received the placebo (M17) (Fig 2F). This finding suggests that treatment with probiotics may stimulate receptors responsible for modulating eosinophilia and SIgA levels, which have a role in parasite control in single-infected mice. We also assessed, *Ascaris’* potential to modulate the expression of these receptors after multiple exposures (S3 Fig).

### Eosinophils are required for mucosal SIgA production and parasite burden control during larval ascariasis in mice

**Fig 3:**
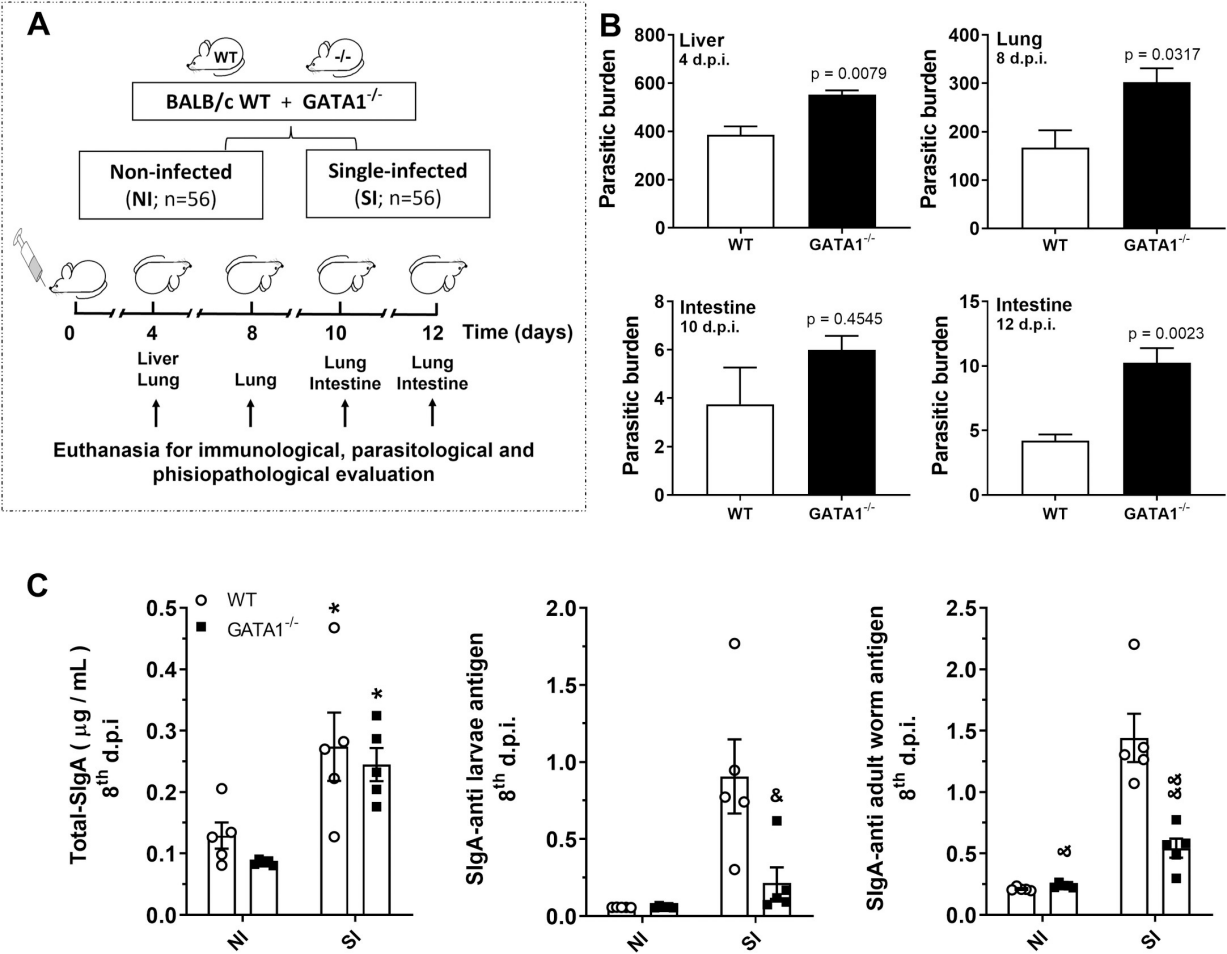
Eosinophils are required for SIgA production and parasite burden control in mice. Schematic representation of the experimental design of a single-infection model using WT BALB/c and GATA1^-/-^ mice (eosinophil deficient) (n = 5) (A). Number of larvae recovered from the liver, lung, and intestine (B) of WT BALB/c and GATA1^-/-^ mice on the 4^th^, 8^th^, 10^th,^ and 12^th^ dpi. Levels of total and *A*. *suum* antigen-specific SIgA from BAL on the 8^th^ dpi (C). T-test was used to assess differences between the groups in (B); one-way ANOVA followed by Tukey´s multiple comparisons test was used to evaluate differences between groups in (C). Differences were considered significant at p ≤ 0.05 and are represented by symbols in the graphs. * represents differences between the single- and non-infected group’s mice of the same strains, and & represents differences between the WT and GATA1^-/-^ mice with the same treatment [1 symbol = p < 0.05], [2 symbols = p < 0.01]. Data are presented as mean ± SEM.

To further investigate the role of eosinophils in SIgA production during larval ascariasis, we evaluated the immune response to *Ascaris* infection in non-eosinophilic mice (GATA1^-/-^) and compared it to that of wild-type mice (WT BALB/c) (Fig 3A). The absence of eosinophils leads to the loss of parasite burden control in GATA1^-/-^ mice (Fig 3B). The role of eosinophils in regulating the levels of total and *A*. *suum* antigen-specific SIgA was established by the increase in eosinophils in WT BALB/c mice BAL, which was not observed in GATA1^-/-^ mice (Fig 3C). BAL from WT BALB/c and GATA1^-/-^ mice showed a significant increase in total SIgA levels on the 8^th^ dpi, however, this increase was *A*. *suum* antigen-specific in only WT BALB/c mice. This demonstrates the importance of eosinophils in the control of parasites (Fig 3B) and reinforces the important role of eosinophils in the modulation of the humoral immune response during *A*. *suum* infection in mice.

### Eosinophils are essential in modulating SIgA production during *Ascaris suum* infection

**Fig 4:**
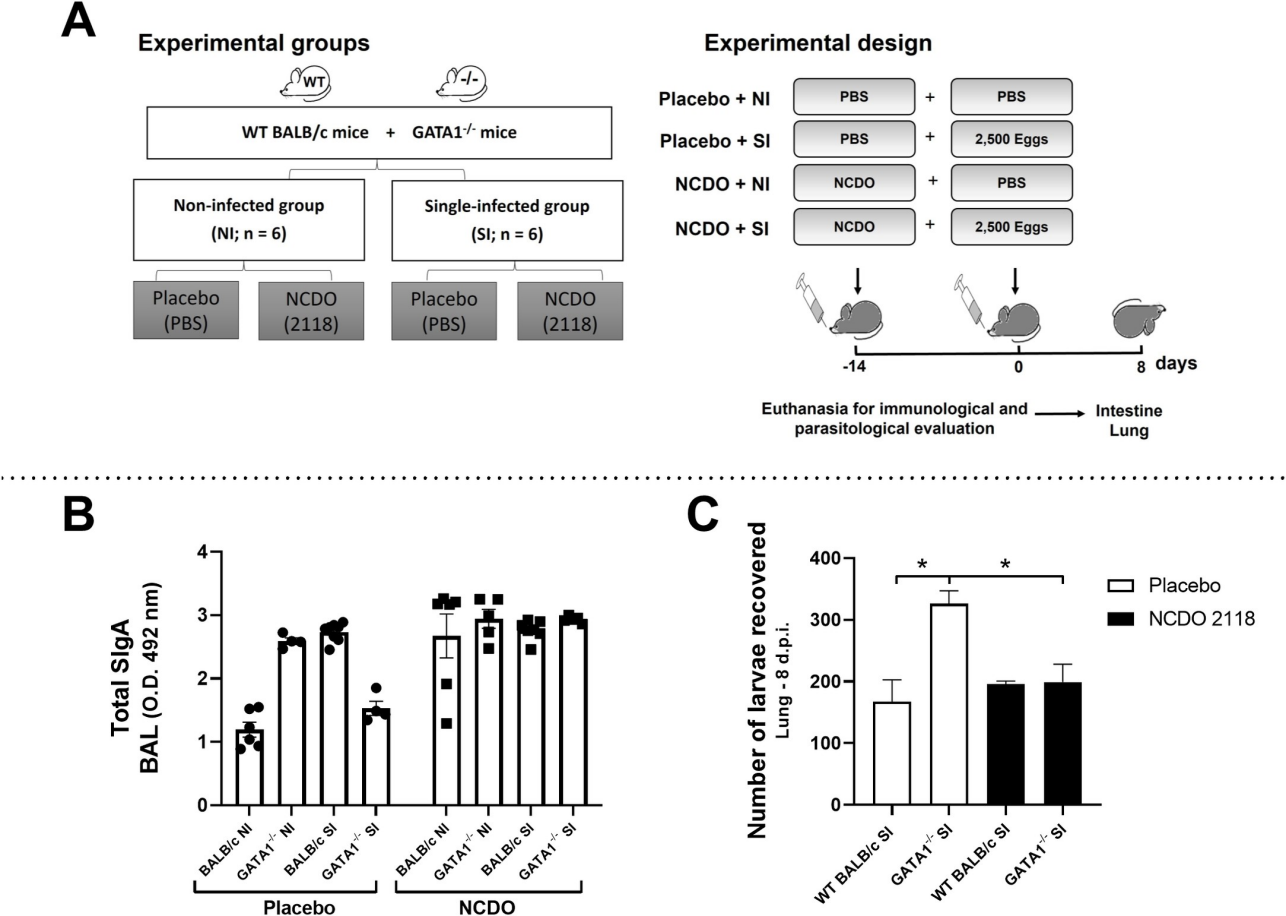
Eosinophils are important in probiotic modulation after infection. Schematic representation of single-infection model using WT BALB/c and GATA1^-/-^ mice after treatment with *Lactococcus lactis* NCDO (2118) (n = 6) (A). Total SIgA levels in BAL on the 22^nd^ day after treatment with probiotic and 8 days after infection (n = 6) (B). Number of larvae recovered from WT BALB/c and GATA1^-/-^ mice lungs (C). Differences between groups were statistically evaluated by the following tests: Two-Way ANOVA followed by Sidak´s multiple comparisons test was used to evaluate differences between groups in (B); One-Way ANOVA followed by Tukey´s multiple comparisons test was used to evaluate differences between groups in (C). Differences were considered significant at p ≤ 0.05 and are represented by symbols in the graph (C) where the * = p < 0.05. Data are presented as mean ± SEM.

To evaluate the role of eosinophils in SIgA modulation after treatment with probiotic *L*. *lactis* (NCDO 2118), GATA1^-/-^ and WT BALB/c mice were treated for 22 days with probiotic and infected with *A*. *suum* (Fig 4A). We found that GATA1^-/-^ NI mice treated with placebo showed high levels of total SIgA in the BAL when compared to WT BALB/c NI and GATA1^-/-^ SI group mice treated with placebo. Interestingly, GATA1^-/-^ SI mice treated with placebo showed lower total SIgA levels (Fig 4B and S2 Table).

We found an increase in total SIgA levels in the WT BALB/c NI group treated with NCDO than in the WT BALB/c NI group treated with placebo. This indicates the efficiency of *L*. *lactis* in inducing the production of total SIgA. Finally, our findings showed that GATA1^-/-^ SI mice treated with NCDO had higher levels of total SIgA than GATA1^-/-^ SI mice treated with placebo, suggesting that probiotic treatment contributes to the maintenance of high levels of total SIgA in the BAL in the absence of eosinophils (Fig 4B and S2 Table).

By analyzing the parasite burden on the 22^nd^ day after treatment with probiotics and 8 dpi (Fig 4C), we found that GATA1^-/-^ SI mice treated with placebo showed higher parasite burden than in WT BALB/c SI mice treated with placebo. Whereas GATA1^-/-^ mice treated with NCDO showed reduced parasite burden than GATA1^-/-^ mice treated with placebo (Fig 4C). Interestingly, no significant differences were observed between wild-type mice.

Together, these results confirm that in the absence of eosinophils, the ability of mice to control parasite burden as well as post-infection SIgA levels reduces. However, the absence of eosinophils did not affect SIgA production under basal conditions or infection, suggesting that eosinophils play different roles under different conditions.

### Eosinophils are associated with inflammation, tissue remodeling, and pulmonary dysfunction caused by larval migration during *A*. *suum* single-infection

**Fig 5:**
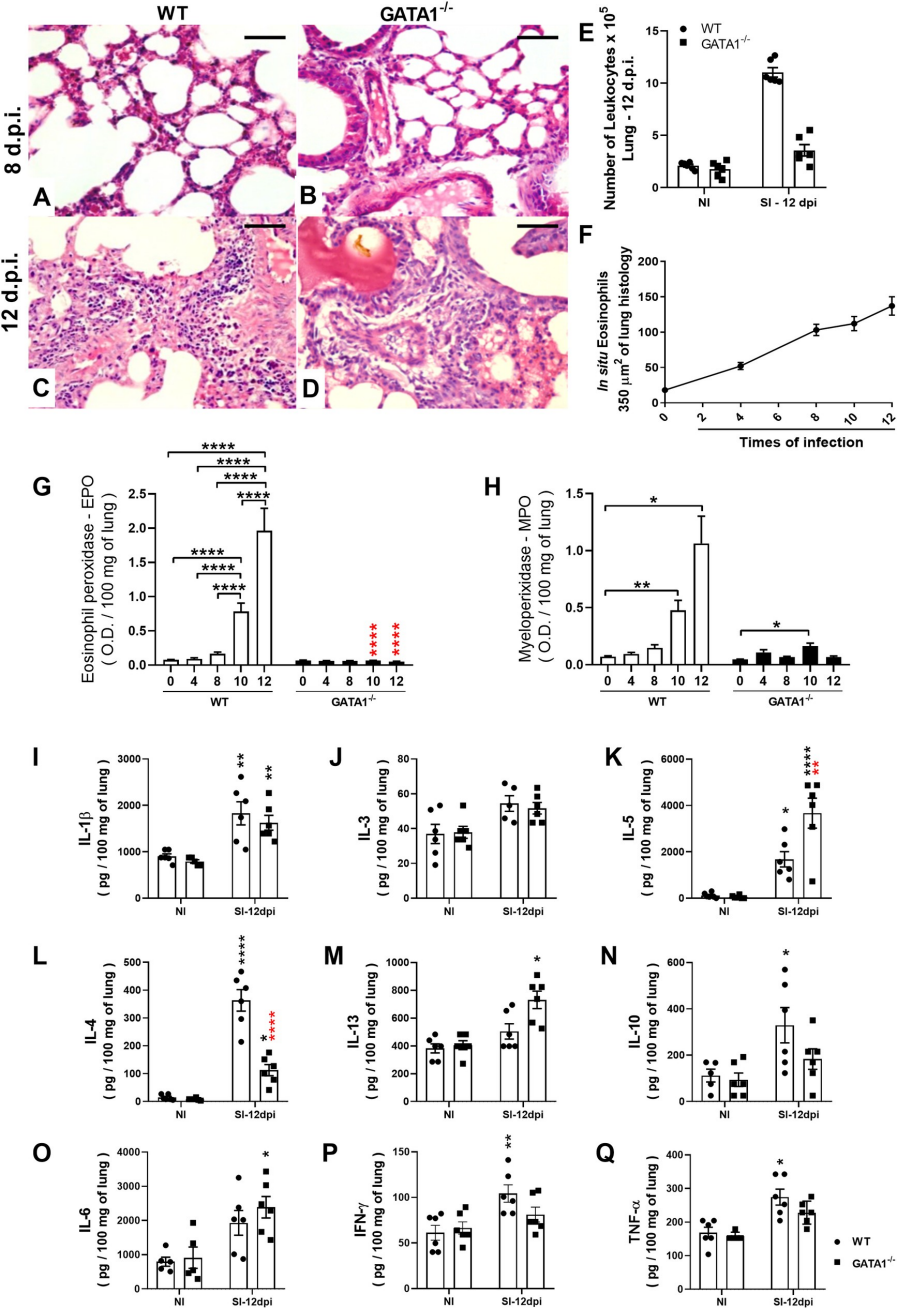
Eosinophils contribute to pulmonary inflammation during *A*. *suum* single-infection in mice. Histopathological analysis of the lesion caused by larval ascariasis and lung inflammation in WT BALB/c and GATA1^-/-^ mice on the 8^th^ and 12^th^ dpi (n = 6). Representative microphotography of lung tissue stained with H&E (Bar scale = 50 μm) (A-D). Number of total leukocytes recovered from BAL on the 8^th^ dpi (n = 6) (E). Number of eosinophils *in situ* in the lung at 0, 4, 8 and 12 dpi (n = 5) (F). Optical density representing Eosinophil peroxidase (EPO) and neutrophil myeloperoxidase (MPO) activity in the lung at 0, 4, 8, and 12 dpi (n = 12) (G and H). Levels of cytokines in the lung at the 12^th^ dpi (n = 6) (I—Q). Data are represented as mean ± SEM. One-way ANOVA followed by Tukey´s multiple comparisons test was used to evaluate differences between groups (G, I, J, K, L, N, O, and P). Kruskal-Wallis test followed by Dunn´s multiple comparisons test was used to evaluate differences between groups (H, M, and Q). The p values are represented by symbols in the graphs wherein * represents differences between the non-infected group, * represents differences between same groups in the 8 dpi, # represents differences between groups from different strains that received the same treatment. [1 symbol = p < 0.05], [2 symbols = p < 0.01], [3 symbols = p < 0.001] and [4 symbols = p < .0001].

In addition to contributing to the control of parasite burden, eosinophils are important in pulmonary pathophysiology during larval ascariasis, and consequently, fibrosis modulation [10]. Therefore, the role of eosinophils in the modulation of pulmonary inflammation following *Ascaris* infection was assessed through the number of innate cells, cytokine profile, and eosinophil and neutrophil activity in lung tissue between non-infected and single-infected WT BALB/c and GATA1^-/-^ mice. Qualitative analyses of leukocyte influx into the lungs during larval migration in WT BALB/c mice revealed neutrophils to the most abundant at the peak of larval migration (8^th^ dpi). However, eventually (12^th^ dpi), the inflammatory infiltrate was more pronounced, mostly composed of mononuclear cells and eosinophils (Fig 5A). Similarly, quantitative morphometric analyses confirmed that *Ascaris* larval migration induced significant pulmonary cell infiltration on the 8^th^ and 12^th^ dpi (243 ± 48 vs. 484 ± 95cells/74588.6 μm^2^ of tissue) (Fig 5B), with progressive accumulation of eosinophils up to the 12^th^ dpi (Fig 5C). In contrast, GATA1^-/-^ mice showed a significant reduction in the number of leukocytes in the airways (Fig 5D) and a reduction in inflammatory cell accumulation in the lung tissues at 12^th^ dpi than in WT BALB/c mice (330 ± 20 vs. 484 ± 95cells/74588.6 μm^2^ of tissue).

The results revealed that larval migration in lungs induced a marked increase in cytokine and granule production in the pulmonary parenchyma on the 12^th^ dpi in WT BALB/c mice than in non-infected mice (Fig 5E–5Q). However, GATA1^-/-^ mice showed a marked ablation of EPO (Fig 5G) and reduced MPO levels (Fig 5F), mainly on the 12^th^ dpi than in single-infected WT BALB/c mice. In addition, at the 12^th^ dpi, there was a significant increase in IL-4, IL-10, IFN-γ, and TNF-α levels (Fig 5L, 5N, 5P, and 5Q) in single-infected WT BALB/c group but not in single-infected GATA1^-/-^ mice. Whereas an increase in IL-5 and IL-13 was observed mainly in GATA1^-/-^ SI mice (Fig 5K and 5M). Collectively, our data support that eosinophils contribute to lung inflammation during larval ascariasis in mice.

**Fig 6:**
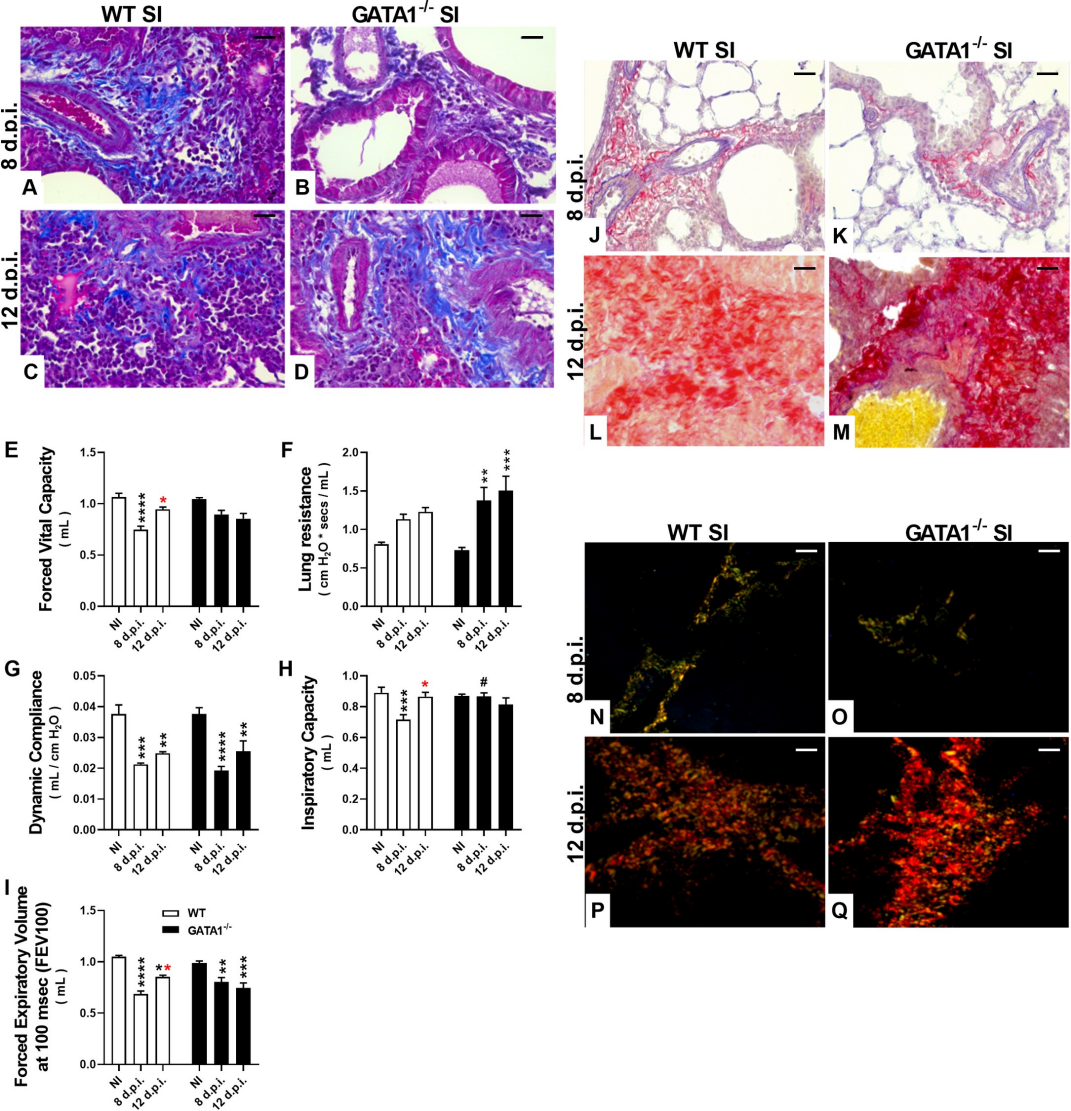
Pulmonary mechanical dysfunction and fibrosis induced by larval ascariasis in the lungs. Representative photomicrographs of single-infected WT BALB/c and GATA1^-/-^ mice (n = 5): Fibrosis analysis using Masson’s trichrome technique (stains collagen fibers blue) and Picrosirius Red (stains collagen fibers red) performed at the 8^th^ dpi (A, J and B, K) showing normal histological appearance with few collagen fibers and at 12^th^ dpi (C, L, and D, M) showing increase in collagen deposition in single-infected WT BALB/c and GATA1^-/-^ mice. Histological analysis at the 12^th^ dpi using Picrosirius Red with polarized light showing increased deposition of type I collagen fibers (stained red, P and Q), distinct from type III collagen observed at the 8^th^ dpi (stained green and yellow, N and O). Assessment of invasive pulmonary spirometry of *Ascaris* single-infected WT BALB/c and GATA1^-/-^ mice through the following parameters: Forced Vital Capacity, FVC (E), Lung Resistance, Rl (F), Dynamic Compliance, Cdyn (G), Inspiratory Capacity, IC (H), Forced Expiratory Volume at 100 msec, FEV100 (I). Data are represented as mean ± SEM. Significant differences between GATA1^-/-^ and WT BALB/c groups at the 12^th^ dpi are represented in the graph by p values. P values are represented by symbols in the graphs wherein * represents differences between non-infected groups from the same strain, * represents differences between the single-infected groups from the same strain, # represents differences between groups from different strains that received the same treatment. One-way ANOVA followed by Tukey´s multiple comparisons test was used to evaluate differences between groups. [1 symbol = p < 0.05], [2 symbols = p < 0.01], [3 symbols = p < 0.001] and [4 symbols = p < 0.0001]. Bar scale = 50 μm.

Despite the increased levels of mediators, such as IL-4 and IL-13, we further investigated tissue fibrosis and pulmonary mechanical dysfunction. Histological analysis performed at the 8^th^ dpi showed normal collagen levels in the lung tissue of *A*. *suum* single-infected GATA1^-/-^ and WT BALB/c mice, while tissue fibrosis was observed at the 12^th^ dpi in both groups. (Fig 6A–6D). Tissue remodeling-induced larval migration lead to pulmonary dysfunction, which was more pronounced in GATA1^-/-^ mice than in WT BALB/c mice. We observed that larval migration through the lungs induced a significant reduction in pulmonary volumes, as depicted by (FVC), inspiratory capacity (IC), and forced expiratory volume (FEV100) at 8^th^ dpi with partial recovery at 12^th^ dpi in WT BALB/c mice (Fig 6E, 6H and 6I); however, GATA1^-/-^ mice showed a marked decline in pulmonary volumes at 12th dpi (Fig 6E and 6I). Similarly, increase in lung resistance (Rl) and dynamic compliance (Cdyn) decline revealed that GATA1^-/-^ infected mice¸ compared to WT infected mice, showed a progressive loss in pulmonary elasticity (Fig 6F and 6G), suggesting that excessive larval migration could induce increased tissue damage and trigger excessive fibrosis.

To further characterize the predominance of collagen types in lung tissue, we used Masson’s trichrome staining and Picrosirius Red, with or without polarized light. We observed a normal histological appearance with few collagen fibers in both WT BALB/c and GATA1^-/-^ mice groups at the 8^th^ dpi (Fig 6J and 6K). Interestingly, an increase in collagen deposition was observed in single-infected WT BALB/c mice at the 12^th^ dpi (Fig 6L) and was even more pronounced in GATA1^-/-^ mice at the 12^th^ dpi (Fig 6M). However, analysis with polarized light on Picrosirius Red showed increased deposition of type I collagen at the 12^th^ dpi (stained red, Fig 6P and 6Q) instead of type III collagen observed at the 8^th^ dpi (stained green, Fig 6N and 6O).

### Eosinophils contribute to parasite burden control and SIgA production during larval ascariasis after multiple exposures

**Fig 7:**
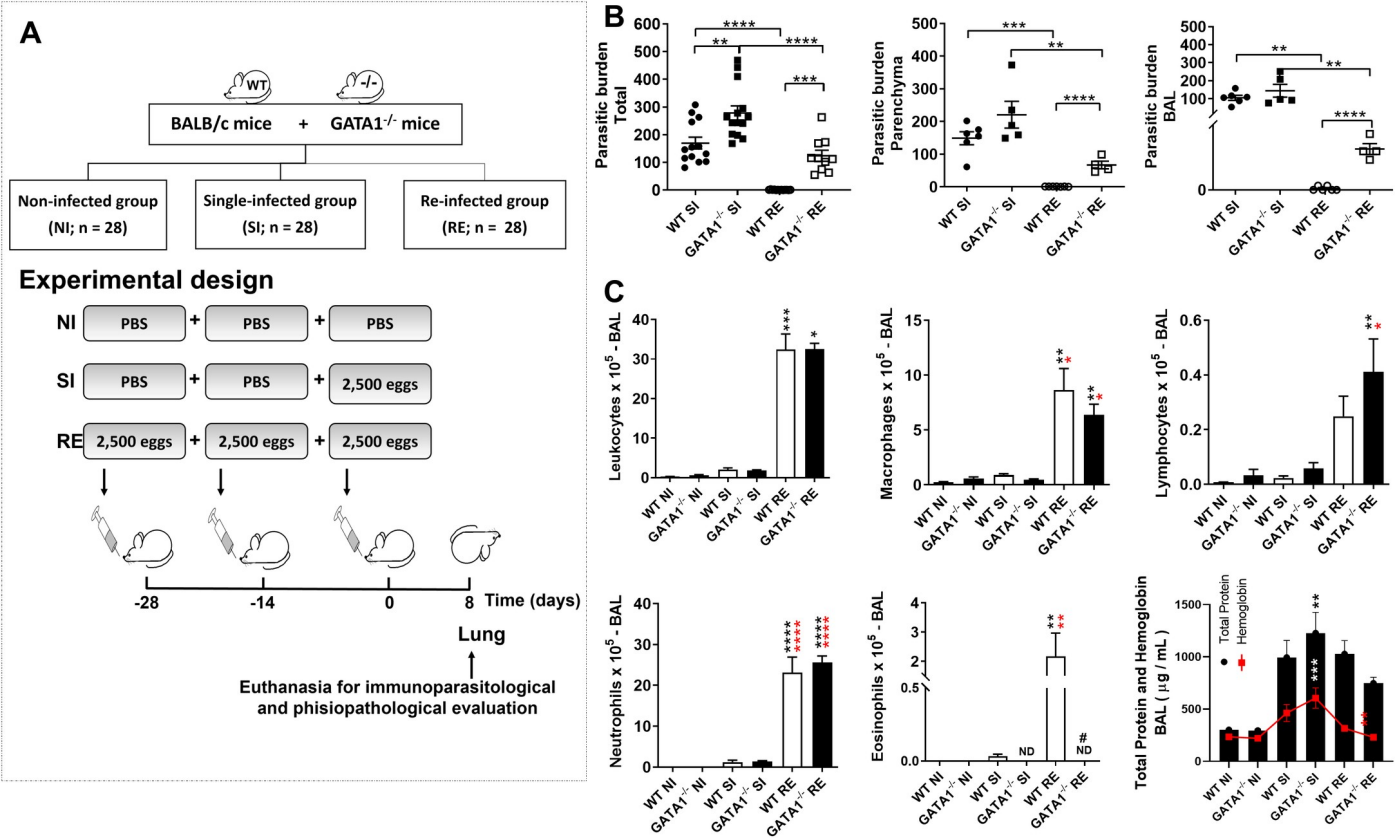
Eosinophils contribute to parasite burden control and SIgA production during larval ascariasis after multiple exposures. Experimental design of *A*. *suum* multiple infections (A). Number of larvae in WT BALB/c and GATA1^-/-^ mice lung parenchyma and bronchoalveolar lavage (BAL) (n = 14) (B). Number of total leukocytes and their subpopulations, namely, lymphocytes, macrophages, neutrophils, eosinophils, and total protein and hemoglobin levels in BAL (n = 6) (C). All immunological and parasitological parameters were evaluated at the 8^th^ dpi. Data are represented as mean ± SEM. Significant differences (p < 0.05) between are represented in the graph by p symbols. * represents differences between non-infected groups from the same strain, * represents differences between the single-infected groups from the same strain, # represents differences between groups from different strains that received the same treatment. [1 symbol = p < 0.05], [2 symbols = p < 0.01], [3 symbols = p < 0.001] and [4 symbols = p < .0001].

Having highlighted the importance of SIgA in controlling parasite burden as well as its correlation with the number of eosinophils, we used a re-infection model previously established by our group [9], which provided evidence of protection after re-infection. Thus, in the experimental design, we compared single-infected (SI) WT BALB/c and GATA1^-/-^ groups to re-infected (RE) WT BALB/c and GATA1^-/-^ groups (Fig 7A) at the 8^th^ dpi, which correspond to the pulmonary phase of *Ascaris* larval migration. Initially, we observe decreased parasite burden in WT BALB/c re-infected mice. We found that eosinophil deficiency in GATA1^-/-^ mice in both single- and re-infected groups reduced parasite burden control when compared to the WT BALB/c groups (Fig 7B). Furthermore, parasite numbers in different lung compartments (airways and tissue), showed that more larvae transmigrated into the airways of GATA1^-/-^ than in the WT BALB/c re-infected group (Fig 7B), suggesting that eosinophils may control the parasite cycle in mice.

We found no eosinophils in the BAL in GATA1^-/-^ mice, whereas the WT BALB/c re-infected group showed a significant increase in eosinophils (Fig 7C). In addition, re-infected WT BALB/c and GATA1^-/-^ mice showed a significant increase in the total number of leukocytes, macrophages, neutrophils, and lymphocytes than NI and SI BALB/c and GATA1^-/-^ mice. However, the increase in lymphocyte numbers was more accentuated in the GATA1^-/-^ re-infected group (Fig 7C). BAL evaluation also showed increased levels of total protein and hemoglobin in the airways of single- and re-infected WT BALB/c and GATA1^-/-^ mice but was more pronounced in the GATA1^-/-^ single-infected group (Fig 7C). There was a positive correlation between total protein and hemoglobin levels in the airways (r^2^ = 0.666, p = 0.0001), probably due to hemorrhage caused by larval migration.

**Fig 8:**
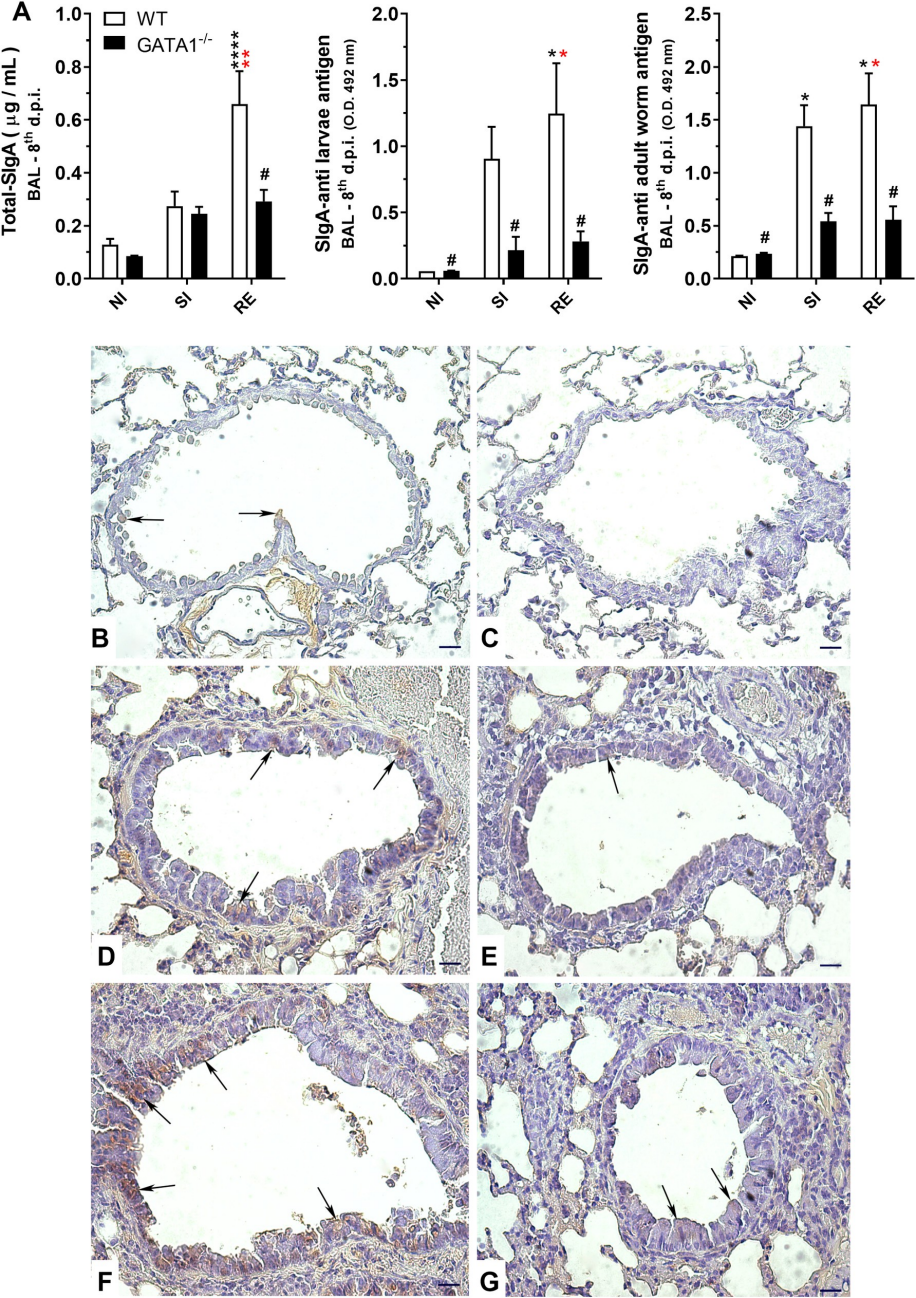
Effect of eosinophil deficiency in IgA production in lungs after larval ascariasis. Total SIgA and *Ascaris*-specifics SIgA levels in bronchoalveolar lavage (BAL) (n = 5) (A). Representative microphotography of IgA immunohistochemistry in lung tissues (B-G) showing immunostained cells predominantly in the peribronchial spaces and the bronchial epithelium. Non-infected WT BALB/c (B), non-infected GATA1^-/-^ (C), single-infected WT BALB/c (D), single infected GATA1^-/-^ (E), re-infected WT BALB/c (F), and re-infected GATA1^-/-^ (G) (n = 6). IgA is marked in brown. Arrows indicate positive markings on the bronchial epithelium. Bar scale = 40 μm).

The relevance of eosinophils and SIgA levels in the lung mucosa and their ability to control parasite burden was more evident in the re-infection model, as in contrast to high levels of total and *A*. *suum* antigen-specific SIgA levels (against antigens from adult worms and larvae of *A*. *suum*) in WT infected mice, the total and specific SIgA levels remained low in eosinophil-deficient mice (GATA1^-/-^) even after re-infection (Fig 8A). To reinforce this relationship, we assessed lung tissue of mice for IgA-positive cells and found *A*. *suum* induced high IgA immunostaining, predominantly in the peribronchial spaces and in the bronchial epithelium to be more prominent in re-infected WT BALB/c mice (Fig 8F) than in single-infected WT BALB/c mice (Fig 8D). The single-infected (Fig 8E) and re-infected GATA1^-/-^ mice (Fig 8G) showed little to no staining.

Furthermore, we measured specific antibodies against *Ascaris-*antigens IgM, IgG1, IgG2A, IgG2B, and IgG3 and found that reinfected WT BALB/c and GATA1^-/-^ mice showed a significant increase in the serum antibodies, indicating that the absence of eosinophils did not compromise specific antibody production (S4 Fig). These data suggest that despite increased production of IgM, IgG1, IgG2A, IgG2B, and IgG3 antibodies, the absence of eosinophils and reduction in SIgA compromised the infection control. Specific IgE levels were not detected.

### Eosinophils are critical for chronic pulmonary inflammation and dysfunction recovery after multiple parasite exposure in mice

**Fig 9:**
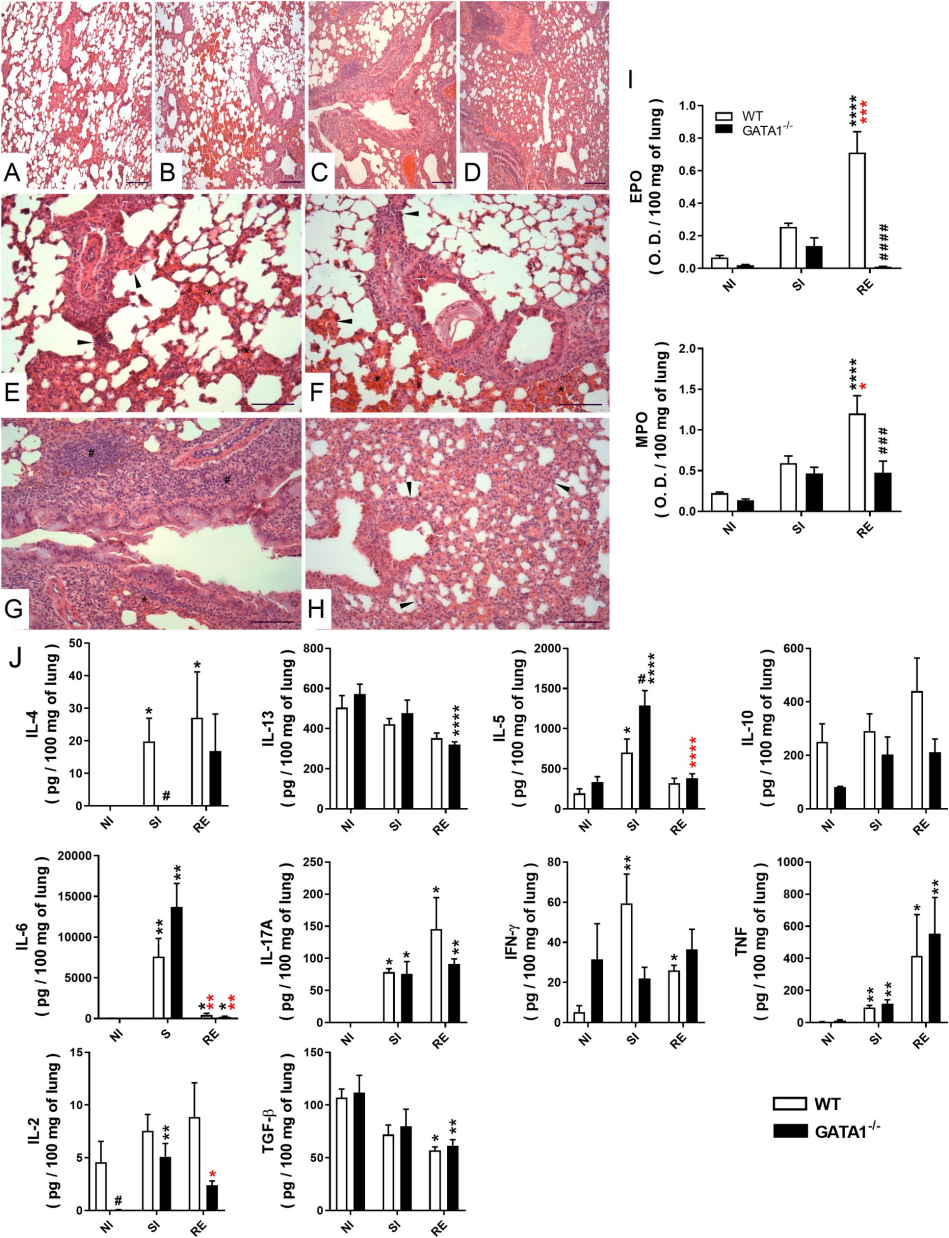
Effect of eosinophil deficiency on chronic pulmonary inflammation induced by multiple *A*. *suum* infections. Representative microphotography of lungs tissues stained with H&E and observed on the 8^th^ dpi at low magnification (Bar scale = 100 μm) (A-D) and at high magnification (Bar scale = 50 μm) (E-H), showing histopathological lesions in the single-infected group of WT BALB/c (A and E) and GATA-/- (B and F) (n = 6), and re-infected WT BALB/c (C and G) and GATA1^-/-^ groups (D and H) (n = 6). Thickened septa are indicated with arrowheads, inflammatory infiltrates are indicated with ‘#’, hemorrhage is indicated with ‘*’. Optical density represents eosinophil peroxidase (EPO) and neutrophil myeloperoxidase (MPO) activity in the lung tissue at the 8^th^ dpi (n = 10) (I). Tissue cytokine profile in the 8^th^ dpi (n = 10) (J). Differences between groups were statistically evaluated using one-way ANOVA followed by Tukey´s multiple comparisons test for the data represented in (I) and the Kruskal-Wallis followed by Dunn’s multiple comparisons test in the data represented in (J). Data are represented as mean ± SEM. P values are represented by symbols in the graphs wherein * represents differences between non-infected groups, * represents differences between single-infected groups, # represents differences between groups from different strains that received the same treatment. [1 symbol = p < 0.05], [2 symbols = 0.01 > p > 0.05], [3 symbols = 0.001 > p > 0.01] and [4 symbols = p < 0.0001].

Evaluation of tissue injury caused by larval migration and lung inflammation revealed that all infected animals (subjected to single and multiple infections) from both mouse strains presented microscopic lesions in the parenchyma characterized by septal thickening, hemorrhage, vascular hyperemia, perivascular edema, and inflammation of the pulmonary parenchyma (Fig 9A–9H). However, these findings were more prominent in re-infected groups regardless of mouse strain, corroborating the results of morphometric evaluations of the lesion area. The lesion area did not differ between groups that received the same treatment, however, lesion characteristics differed between groups. Hemorrhage was more pronounced in single-infected groups from both mouse strains than in the GATA1^-/-^ mice re-infected group, reinforcing that the injuries caused by larval migration are responsible for increased hemorrhage in the lungs. The inflammatory infiltrate was more prominent in the WT BALB/c and GATA1^-/-^ re-infected groups. However, re-infected WT BALB/c mice showed mixed inflammatory infiltrate (with neutrophil and eosinophil predominance). These findings corroborate the finding of EPO and MPO assays that showed a significant increase in eosinophil and neutrophil activity in WT BALB/c RE mice when compared to the other groups (Fig 9I). Single- and re-infected GATA1^-/-^ mice had blunted EPO levels and reduced MPO levels from tissue neutrophils and discrete bronchus-associated lymphoid tissues (BALT) formation.

Th1/Th2/Th17 type cytokines were measured to further characterize lung inflammation (Fig 9J). We found a significant increase in IL-2, IL-4, IL-10, and IFN-γ in the WT BALB/c single- and re-infected groups when compared to non-infected mice, but not in the GATA1^-/-^ single- and re-infected groups. Inflammatory cytokines, such as IL-6, IL-17A, and TNF, were increased in WT BALB/c and GATA1^-/-^ single- and re-infected groups, when compared to the respective non-infected groups. IL-5 levels were significantly increased in the GATA1^-/-^ and WT BALB/c single-infected mice, but more pronounced in GATA1^-/-^ single-infected mice. No differences in IL-13 and TGF-β1 levels were observed between WT and GATA1^-/-^ single- and re-infected mice (Fig 9J).

**Fig 10:**
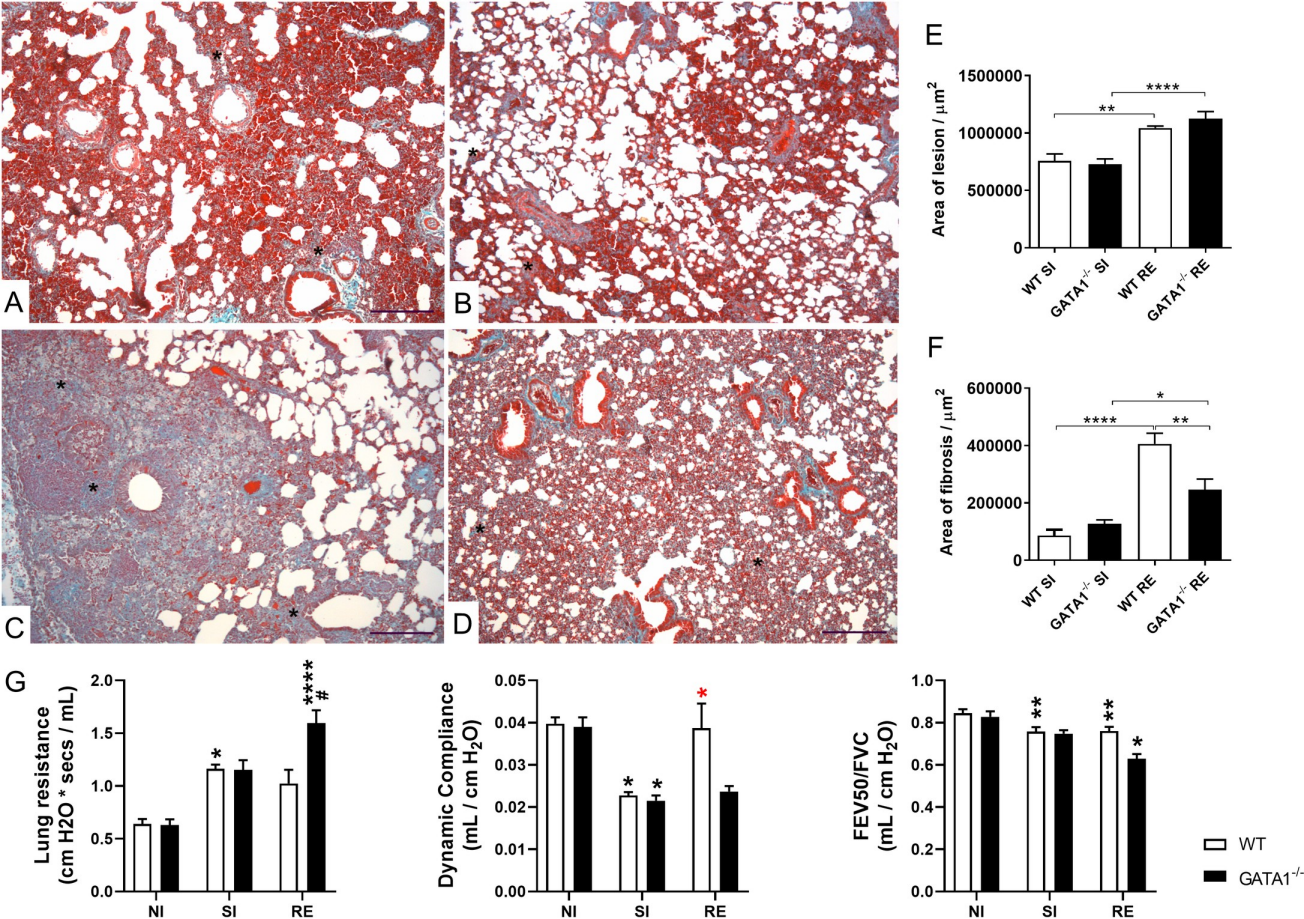
Effect of eosinophil deficiency in lung injury, fibrosis, and dysfunction induced by multiple larval ascariasis. Representative microphotography of fibroplasia/fibrosis histopathological analysis from lung tissues stained with Gomori’s trichrome (A-D) showing lesions and inflammation caused by larval ascariasis in single-infected (A-B) compared to re-infected (C-D) WT BALB/c (A and C) and GATA1^-/-^ (B and D) mice (n = 6). Fibrosis is marked in bluish green. Bar scale = 50 μm. The area of fibrosis is represented by ‘*’. Bar graphs from morphometric analysis of the area of the lesion (E) and fibrosis (F) caused by larval migration and lung inflammation (n = 6). Assessment of lung mechanics in mice after single and multiple infections with *A*. *suum* (G) (n = 6). Data are represented as mean ± SEM. One-way ANOVA followed by Tukey´s multiple comparisons test was used to evaluate differences between groups in (E) and (F). Kruskal-Wallis test followed by Dunn´s multiple comparisons test was used to evaluate differences among groups in (G). Statistical differences are represented by symbols in the graphs, where * represents differences observed between non-infected groups, * represents differences between single-infected groups, # represents differences between groups of different strains that received the same treatment. [1 symbol = p < 0.05], [2 symbols = 0.01 > p > 0.05], [3 symbols = 0.001 > p > 0.01] and [4 symbols = p < 0.0001]. All experiments were analyzed at the 8^th^ dpi.

Next, we evaluated pulmonary fibrosis in WT BALB/c and GATA1^-/-^ mice in the single and re-infected groups (Fig 10). Histopathological evaluation of lung tissue sections stained with Gomori’s trichrome was performed to determine the chronic tissue remodeling and fibrosis induced by *A*. *suum* larvae migration. Fibrosis was evident in some areas of alveolar septa, bronchi, bronchioles, and pulmonary blood vessels, which contributed to the thickening of the alveolar septum in single-infected and re-infected mice from both strains (Fig 10) but was more pronounced in re-infected WT BALB/c mice (Fig 10C and 10F). Re-infected GATA1^-/-^ mice showed larger injury areas than GATA1^-/-^ single-infected mice (Fig 10B, 10D, and 10E). Interestingly, corroborating the findings of morphometric analyses, re-infected WT BALB/c mice exhibited large areas of consolidated fibrosis, causing considerable thickening of the alveolar septa, and compromising a larger number of bronchi, bronchioles, and vessels than that observed in GATA1^-/-^ mice (Fig 10C–10F). Invasive spirometry confirmed pulmonary lesions caused by larval migration, inflammation, and fibrosis, which were reflected in pulmonary mechanical dysfunction (Fig 10G). Re-infected WT BALB/c mice did not show worsening lung resistance and dynamic compliance (Fig 10C, 10F and 10G), however, re-infected GATA1^-/-^ mice showed an increase in lung resistance and decreased dynamic compliance resulting from chronic pulmonary inflammation (Fig 10D–10G).

## Discussion

Ascariasis is the most prevalent neglected tropical disease, affecting approximately 450 million people worldwide [2,4]. In ascariasis, larval migration through the host’s organs leads to tissue injury followed by the induction of innate and adaptive immune responses, which are characterized by the production of Th2 and Th17 cytokines by lymphocytes, inflammatory cell infiltration of mainly neutrophils and eosinophils, and immunoglobulin induction against the parasite [8,9]. In this study, we investigated the role of eosinophils and adaptive immune responses during single or multiple larval ascariasis in mice. Our results collectively highlight the complex network involving innate and humoral immune responses that control the parasite burden during *Ascaris suum* infection in mice, and can be summarized as follows: (i) eosinophilia correlates with mucosal SIgA induction and parasite burden, (ii) TLR2 and TLR4 activation mediates eosinophilia and mucosal SIgA production, controlling parasite burden, (iii) eosinophils are required for SIgA production and parasite burden control; (iv) eosinophils are associated with inflammation, remodeling, and pulmonary dysfunction caused by larval migration, (v) eosinophils contribute to parasite burden control and SIgA production after multiple parasite exposures, and (vi) eosinophils are critical for recovery from chronic pulmonary inflammation and dysfunction after multiple parasite exposures in mice.

Evaluation of immunological and parasitological parameters in this study suggest a collaborative cross-talk of innate and adaptative immune responses during pulmonary larval ascariasis in mice. The association between eosinophils and SIgA creates a protective environment against larval migration, resulting in the control of lung damage and dysfunction due to the decrease in parasite burden. Eosinophil activation, degranulation, and cytokine production are mechanisms that may ascribe protection against ascariasis and other helminthiases. The role of eosinophils in helminth protection, as well as the interaction of SIgA and eosinophils in helminth protection, has been well documented [17,21,32]. SIgA interaction with eosinophils regulates both, eosinophil survival and gene expression [33]. SIgA binding alone can prime eosinophils and increase cytokine production and survival while binding of SIgA immunocomplexes with antigens can completely activate the degranulation and superoxide release effector functions of eosinophils [19], as in the case of IgG binding [34,35]. In addition, it has been reported that eosinophils are important sources of APRIL and IL-6 mediators, which are crucial for plasma cell activation and survival, and the absence of eosinophils, the plasma cells quickly die of apoptosis [20,33]. A study in eosinophil-deficient mice revealed that eosinophils also contribute to maintaining plasma cell survival in both Th1 and Th2 immune responses in mice [19,20].

In addition to eosinophils, other immunological cells such as lymphocytes and macrophages, especially those alternatively activated (M2), are important for protection against helminths, repair of damaged tissues, and immune response modulation during helminth infection [36–38]. Furthermore, helper T cells and macrophages, or factors derived from such cells, participate in the induction of IgA production [39,40]. Corroborating these findings, our data indicated that, in addition to eosinophils, macrophages and lymphocytes may also participate in protection by promoting the production of IgA. However, elucidation of the role of lymphocytes and macrophages was not the focus of this study.

Several protective mechanisms associated with SIgA have been described related to its protective function against helminths. These include potent eosinophil degranulation, helminth larvae entrapment, phagocytosis mediated by alternative complement cascade activation, and neutralization of proteins secreted/excreted by the parasite [7]. Although recent studies have shown that eosinophils are not necessary to maintain IgA levels in the absence of infection [41–43], eosinophil-deficient mouse models show a reduction in the number of plasma cells, IgA and SIgA levels in the intestinal lamina propria [32,44–46].

It is hypothesized that the presence of innate SIgA at the site of infection is a natural protective factor that hampers larval migration. In our study, the increase in specific and total SIgA concentrations associated with negative correlations between SIgA concentration and parasite burden supports the hypothesis that SIgA plays a protective role in ascariasis and mitigating the damage caused by larval migration. This was established using GATA1^-/-^ mice that showed a significant reduction in SIgA levels in BAL and lung parenchyma and increased parasite burden with consequent pulmonary dysfunction.

Total SIgA production and the presence of leukocytes in the BAL are dependent on the time of infection. Moreover, positive correlations between parasite burden and immunological variables are consistent with BALT biology because, in mice and humans, BALT is only induced by infections [9]. Corroborating these results, we found positive correlations between SIgA in the BAL and markers of preserved pulmonary function, such as dynamic compliance and pulmonary resistance. Thus, as the larvae migrate to the pulmonary parenchyma, BALT formation and consequent cellular and humoral immune responses occur. Higher SIgA levels in the BAL may reduce parasite burden and consequently, the pathologies associated with pulmonary parenchymal migration and tissue remodeling.

Acute inflammation releases numerous mediators and cytokines that result in cell infiltration and subepithelial fibrosis, which are immunopathological processes associated with larval migration in ascariasis [3,32,44–46]. Ascariasis is characterized by diffuse chronic inflammation of the pulmonary parenchyma with an increase in eosinophils, neutrophils, macrophages, and lymphocytes in the parenchyma and airways, highlighting the mixed character of inflammation and pulmonary pathology typically observed in this disease. In addition, the presence of eosinophils after multiple exposures is critical to the pathogenesis of larval ascariasis, as eosinophils regulate and promote the maintenance of alternatively activated macrophages [47]. Thus, they contribute to the expulsion of parasites and promote type 2 pathogenic responses, as well as tissue repair [48]. Tissue remodeling improved respiratory capacity in re-infected WT BALB/c mice, even with increased tissue fibrosis, compared to re-infected GATA1^-/-^ mice, with a higher parasite burden. This was probably due to the severity of tissue damage observed in mice due to a large number of larvae migrating through the organs. In this regard, our results improve our understanding of the role of eosinophils and SIgA in larval ascariasis, although more studies are needed to elucidate the mechanisms involved in the development of resistance to primary infection and re-infection.

Finally, the greater susceptibility to *A*. *suum* infection in TLR2- and TLR4-deficient mice was associated with a decrease in eosinophils and production of total and antigen-specific SIgA antibodies in the pulmonary mucosa. This suggests that the increase in eosinophils is dependent on TLR2 and TLR4 sensing and eosinophils modulate SIgA levels, mainly in re-infected animals. Besides, similar results have been obtained with different parasites; for example, a study with *Taenia crassiceps* showed that TLR2 appears is essential for limiting infection during experimental cysticercosis, as TLR2 signaling pathways are involved in the recognition and subsequent activation of the innate immune system and production of inflammatory cytokines [49]. Experimental infections with *Schistosoma* sp. showed that TLR2 is involved in modulating the immune response against soluble egg antigen [50,51], which reduces immunopathology, despite favoring the persistence of infection. Moreover, experimental infections revealed the role of TLR4 in protective immunity against larvae of *Onchocerca volvulus* [52] and *Strongyloides stercoralis* [53].

TLR is known to be expressed on the cell surface of a large variety of cells such as dendritic cells, macrophages, B lymphocytes, eosinophils, and lung epithelium cells [54]. Helminths have a wide variety of glycoproteins and glycolipids that bind to TLRs on the host cell surface and activate or modulate the immune response mediated by such receptors [55]. It was demonstrated that *Taenia crassiceps* carbohydrates (TCHO) having repetitive structure characteristic of PAMPs, can link to TLR4 and, to a lesser extent, TLR2; the interaction of TCHO with TLR4 induces IL-6 expression in naïve murine macrophages [49]. A similar mechanism may occur in eosinophils triggered by helminth molecules, probably inducing IL-5 expression, a pleiotropic cytokine that can function as a growth factor for the B-1 cell subset associated with autoantibody production [56], as well as induce B cells to switch to IgA [57], and B cell IgA^+^ differentiation [58].

Proteomic evaluation of hepatic resistance to larval ascariasis revealed upregulation of genes related to innate and adaptive immune responses in *Ascaris*-infected mice [59]. These genes may act as markers of danger-associated molecular patterns (DAMPs) interacting with TLR4 like that observed in asthma [60,61]. Increased TLR2 and TLR4 expression in circulating B-cells during helminth infection has already been demonstrated, reflecting systemic exposure to the microbial ligands [62]. Therefore, a possible mechanism linking TLR activation with SIgA production has already been described [54]. A third signal for efficient naïve B cell proliferation, isotypic exchange for IgG and IgA, and differentiation of antibody-secreting cells has also been shown. A direct signal may be delivered by direct TLR activation in the endosomal compartment or on the plasma membrane of B cells. In addition, the interaction of cytokines produced by TLR-activated dendritic cells may be an indirect signal [63]. TLR4 activation, as well as the presence of tissue damage, induces a considerable increase in polyreactive antibodies [64–66]. Furthermore, DAMP signaling induces somatic hypermutation and leads to an increased antigenic affinity for polyreactive antibodies [67,68]. This process seems to be due to the ambivalent action of this immunoglobulin, as it allows SIgA to discriminate and act against potentially harmful pathogens while acting as an anti-inflammatory molecule in the mucosal homeostasis process [69].

With our growing understanding of intestinal mucosa homeostasis, the existence of an intricate network between microbiota composition, development of immunity, and the mechanisms of susceptibility/resistance to helminth infections is becoming more evident. The presence of helminths in the intestinal mucosa may modify the composition and abundance of bacterial species that make up the intestinal microbiota. Similarly, variations in microbiota composition may alter the susceptibility of the host. This study showed that one of the resistance mechanisms against ascariasis is dependent on TLR2 and TLR4 signaling and the presence of eosinophils, which results in SIgA production.

The role of probiotics in modulating the immune response has been widely discussed. A genomic study associated with in vitro and in vivo experiments demonstrated that the use of the probiotic *Lactobacillus delbrueckii subsp*. *lactis* (CIDCA 133) induced increased expression of TLR2, TLR4, and MyD88 genes, with subsequent production of cytokines [70]. Similar results were found with the use of the probiotics *Lacticaseibacillus casei* and *Saccharomyces boulardii* [71,72].

Oral administration of the probiotic *Lactobacillus gasseri* (SBT 2055) in mice caused an increase in IgA levels and the rate of IgA^+^ cell populations in Peyer’s patches and the lamina propria of the small intestine, as well as in cultures of B cells and dendritic cells derived from bone marrow (BMDC). The addition of anti-TLR2 antibodies inhibits probiotic-induced IgA production [73].

We demonstrated that a diet containing probiotic *Lactococcus* induced a considerable increase in the number of eosinophils in the pulmonary mucosa, as well as the production of total SIgA, specific parasitic immunoglobulin, and an absolute number of cell subpopulations in the BAL. These findings suggest that the probiotic bacteria *L*. *lactis* (NCDO 2118) acts as an adjuvant in inducing a mucosal immune response against *A*. *suum* larvae.

Our findings corroborate the data by FitzPatrick et al. (2020), who described high levels of total SIgA under natural conditions and without infection in the absence of eosinophils [41]. However, we also found that eosinophils are essential for the maintenance of high levels of this immunoglobulin after infection with *A*. *suum*, corroborating the findings of previous studies [32,44–46]. We also noticed high levels of antibodies after infection in GATA1^-/-^ mice, which may have contributed to the reduced parasite burden in these animals.

Although Luerce et al. (2014) demonstrated that *L*. *lactis* (NCDO 2118) did not alter SIgA production under physiological conditions in WT C57BL/6 mice (2, 3, and 4 days after treatment) [22], our results showed that after 22 days of treatment, there was a significant increase in SIgA in BALB/c and GATA1^-/-^ mice. These findings are supported by previous studies that demonstrated that during intestinal parasitic infections, additional eosinophils are recruited into the intestinal tract [74,75]. Moreover, a local eosinophilic response may also induce an increased number of eosinophils in remote sites [76]. Although some variations in basal SIgA levels have been observed in non-infected mice before, the literature suggests that variations in the native microbiota are natural [41]. It has also been reported that WT BALB/c and WT C57BL/6 mice strains differ in polyreactive IgA abundance and specificity, as well as in microbiota diversity [77].

Innate and adaptive immune responses are intrinsically connected, and their cross-talk is very important for homeostasis and is crucial in dealing with helminthiasis. Taken together, our results highlight the importance of eosinophils in mediating IgA production in mucosal sites, tissue inflammation, and remodeling, as well as controlling parasite burden during experimental larval ascariasis in mice. In conclusion, this study reinforces the known essential nature of eosinophils by coordinating multiple factors involved in the immune response to helminth infections. In addition, we postulate that stimulation of TLR may contribute to controlling parasitic burden via eosinophils and SIgA.

## Supporting information

S1 Fig Negative control of the immunohistochemical reaction. Histological lung sections of re-infected WT BALB/c mice.
(TIF)

S2 Fig Antigen-specific antibodies IgM, IgG1, IgG2A, IgG2B, and IgG3 were measured by ELISA in WT BALB/c and WT C57BL/6 mice at different times of infection. Data are represented as mean ± SEM. The Kruskal-Wallis test followed by Dunn´s multiple comparisons test was used to evaluate differences between groups (A-D). Significant differences (p < 0.05) are represented in the graph by symbols. * represents differences between the evaluated time and the previous times in the same strains of mice; # represents the differences between different strains at the same time of infection. [1 symbol = p < 0.05], [2 symbols = p < 0.01], and [3 symbols = p < 0.001].
(TIF)

S3 Fig Relative expression of TLR-2 and TLR-4 mRNA. RT-qPCR data were obtained using the cDNA of non-infected (NI) and single-infected (SI) BALB/c and C57BL/6 mice and re-infected (RE) BALB/c mice (n = 5 / group). The relative expression of TLR-2 and TLR-4 mRNA was normalized to the reference gene β-actin and the fold change was calculated using the 2^(-ΔΔCt)^ method. The fold change was expressed as mean ± standard deviation for all groups. The Mann-Whitney test was used to evaluate the differences between the groups (A and D). One-way ANOVA followed by Tukey´s multiple comparisons test was used to evaluate the differences between groups (B and E). Statistical differences are represented by symbols in the graphs, where * represents p < 0.05, ** represents p < 0.01. All experiments were performed on the 8th dpi. RT-qPCR products were analyzed using 2% agarose gel electrophoresis. The 191 bp and 249 bp bands amplicons correspond to the amplified fragments of the TLR-2 (C) and TLR-4 (F) cDNA, respectively. bp: base pairs.
(TIF)

S4 Fig Total SIgA and antigen-specific antibodies IgM, IgG1, IgG2A, IgG2B, and IgG3 were measured by ELISA in WT BALB/c and GATA1-/- mice after single- and re-infection. P values are represented by symbols in the graphs wherein * represents differences between the non-infected groups of the same strain, * represents differences between the single-infected groups of the same strain, # represents differences between groups from different strains that received the same treatment. One-way ANOVA followed by Tukey´s multiple comparisons test was used to evaluate differences between groups (A-F). [1 symbol = p < 0.05], [2 symbols = p < 0.01], [3 symbols = p < 0.001], and [4 symbols = p < 0.0001].
(TIF)

S1 Table Statistical differences in the total number of leukocytes and their cell subpopulations in bronchoalveolar lavage fluid at different times of infection. Significant p-values (p < 0.05) are indicated in bold. One-way ANOVA followed by Tukey´s multiple comparisons test was used to evaluate the differences between times.
(DOCX)

S2 Table Statistical differences in total SIgA levels in the bronchoalveolar lavage (BAL) 8 dpi with *Ascaris suum* and after 22 days of treatment with 1 × 109 CFU/mL of *Lactococcus lactis* (NCDO (2118) or placebo (PBS). Groups that presented higher levels of SIgA are shown on the left. Statistical differences are indicated by p-values and symbols in bold. Two-way ANOVA followed by Sidak’s multiple comparisons test was used to evaluate differences between groups.
(DOCX)

S3 Table Statistical differences in total SIgA levels in the intestine lavage 8 dpi with *Ascaris suum* and after 22 days of treatment with 1 × 109 CFU/mL of *Lactococcus lactis* (NCDO (2118)) or placebo. Two-way ANOVA followed by Sidak’s multiple comparisons test was used to evaluate differences between groups.
(DOCX)

## References

[1] Lafferty EI, Qureshi ST, Schnare M. The role of toll-like receptors in acute and chronic lung inflammation. Journal of Inflammation. 2010;7: 57. doi: 10.1186/1476-9255-7-57 21108806 PMC3003652

[2] Vos T, Barber RM, Bell B, Bertozzi-Villa A, Biryukov S, Bolliger I, et al. Global, regional, and national incidence, prevalence, and years lived with disability for 301 acute and chronic diseases and injuries in 188 countries, 1990–2013: A systematic analysis for the Global Burden of Disease Study 2013. The Lancet. 2015;386: 743–800. doi: 10.1016/S0140-6736(15)60692-4 26063472 PMC4561509

[3] Else KJ, Keiser J, Holland C v., Grencis RK, Sattelle DB, Fujiwara RT, et al. Whipworm and roundworm infections. Nature Reviews Disease Primers. 2020;6: 44. doi: 10.1038/s41572-020-0171-3 32467581

[4] James SL, Abate D, Abate KH, Abay SM, Abbafati C, Abbasi N, et al. Global, regional, and national incidence, prevalence, and years lived with disability for 354 Diseases and Injuries for 195 countries and territories, 1990–2017: A systematic analysis for the Global Burden of Disease Study 2017. The Lancet. 2018;392. doi: 10.1016/S0140-6736(18)32279-7 30496104 PMC6227754

[5] Berek C. Eosinophils: important players in humoral immunity. Clinical & Experimental Immunology. 2016;183: 57–64. doi: 10.1111/cei.12695 26291602 PMC4687508

[6] Rothenberg ME, Hogan SP. THE EOSINOPHIL. Annual Review of Immunology. 2006;24: 147–174. doi: 10.1146/annurev.immunol.24.021605.090720 16551246

[7] Shamri R, Xenakis JJ, Spencer LA. Eosinophils in innate immunity: An evolving story. Cell and Tissue Research. 2011;343: 57–83. doi: 10.1007/s00441-010-1049-6 21042920 PMC3679536

[8] Gazzinelli-Guimarães PH, Gazzinelli-Guimarães AC, Silva FN, Mati VLT, Dhom-Lemos L de C, Barbosa FS, et al. Parasitological and immunological aspects of early Ascaris spp. infection in mice. International Journal for Parasitology. 2013;43: 697–706. doi: 10.1016/j.ijpara.2013.02.009 23665127

[9] Nogueira DS, Gazzinelli-Guimarães PH, Barbosa FS, Resende NM, Silva CC, de Oliveira LM, et al. Multiple Exposures to Ascaris suum Induce Tissue Injury and Mixed Th2/Th17 Immune Response in Mice. PLoS Neglected Tropical Diseases. 2016;10: 1–19. doi: 10.1371/journal.pntd.0004382 26814713 PMC4729520

[10] Oliveira FMS, Matias PHDP, Kraemer L, Gazzinelli-Guimarães AC, Santos FV, Amorim CCO, et al. Comorbidity associated to ascaris suum infection during pulmonary fibrosis exacerbates chronic lung and liver inflammation and dysfunction but not affect the parasite cycle in mice. PLoS Neglected Tropical Diseases. 2019;13. doi: 10.1371/journal.pntd.0007896 31765381 PMC6901262

[11] Halim TYF, Steer CA, Mathä L, Gold MJ, Martinez-Gonzalez I, McNagny KM, et al. Group 2 innate lymphoid cells are critical for the initiation of adaptive T helper 2 cell-mediated allergic lung inflammation. Immunity. 2014;40: 425–435. doi: 10.1016/j.immuni.2014.01.011 24613091 PMC4210641

[12] Gazzinelli-Guimaraes PH, de Queiroz Prado R, Ricciardi A, Bonne-Année S, Sciurba J, Karmele EP, et al. Allergen presensitization drives an eosinophil-dependent arrest in lung-specific helminth development. Journal of Clinical Investigation. 2019;129: 3686–3701. doi: 10.1172/JCI127963 31380805 PMC6715365

[13] Weatherhead JE, Porter P, Coffey A, Haydel D, Versteeg L, Zhan B, et al. Ascaris larval infection and lung invasion directly induce severe allergic airway disease in mice. Infection and Immunity. 2018;86: 1–12. doi: 10.1128/IAI.00533-18 30249744 PMC6246907

[14] Wolterink RGJK, KleinJan A, van Nimwegen M, Bergen I, de Bruijn M, Levani Y, et al. Pulmonary innate lymphoid cells are major producers of IL-5 and IL-13 in murine models of allergic asthma. European Journal of Immunology. 2012;42: 1106–1116. doi: 10.1002/eji.201142018 22539286

[15] Bernink JH, Germar K, Spits H. The role of ILC2 in pathology of type 2 inflammatory diseases. Current Opinion in Immunology. 2014;31: 115–120. doi: 10.1016/j.coi.2014.10.007 25459003

[16] Licona-Limón P, Kim LK, Palm NW, Flavell RA. TH2, allergy and group 2 innate lymphoid cells. Nature Immunology. 2013;14: 536–542. doi: 10.1038/ni.2617 23685824

[17] Henderson NG, Stear MJ. Eosinophil and IgA responses in sheep infected with Teladorsagia circumcincta. Veterinary Immunology and Immunopathology. 2006;112: 62–66. doi: 10.1016/j.vetimm.2006.03.012 16684572

[18] Jung Y, Wen T, Mingler MK, Caldwell JM, Wang YH, Chaplin DD, et al. IL-1β in eosinophil-mediated small intestinal homeostasis and IgA production. Mucosal Immunology. 2015;8: 930–942. doi: 10.1038/mi.2014.123 25563499 PMC4481137

[19] Chu VT, Beller A, Rausch S, Strandmark J, Zänker M, Arbach O, et al. Eosinophils Promote Generation and Maintenance of Immunoglobulin-A-Expressing Plasma Cells and Contribute to Gut Immune Homeostasis. Immunity. 2014;40: 582–593. doi: 10.1016/j.immuni.2014.02.014 24745334

[20] Chu VT, Fröhlich A, Steinhauser G, Scheel T, Roch T, Fillatreau S, et al. Eosinophils are required for the maintenance of plasma cells in the bone marrow. Nature Immunology. 2011;12: 151–159. doi: 10.1038/ni.1981 21217761

[21] Kanobana K, Ploeger HW, Vervelde L. Immune expulsion of the trichostrongylid Cooperia oncophora is associated with increased eosinophilia and mucosal IgA. Int J Parasitol. 2002;32: 1389–1398. doi: 10.1016/s0020-7519(02)00132-7 12350374

[22] Luerce TD, Gomes-Santos AC, Rocha CS, Moreira TG, Cruz DN, Lemos L, et al. Anti-inflammatory effects of Lactococcus lactis NCDO 2118 during the remission period of chemically induced colitis. Gut Pathogens. 2014;6: 1–11. doi: 10.1186/1757-4749-6-1 25110521 PMC4126083

[23] Zurita-turk M, Souza BM, Castro CP de, Pereira VB, Pecini V, Preisser TM, et al. Attenuation of intestinal inflammation in IL- 10 deficient mice by a plasmid carrying Lactococcus lactis strain. BMC Biotechnology2. 2020;20: 1–12. doi: 10.1186/s12896-020-00631-0 32703192 PMC7379781

[24] Boes J, Eriksen L, Nansen P. Embryonation and infectivity of Ascaris suum eggs isolated from worms expelled by pigs treated with albendazole, pyrantel pamoate, ivermectin or piperazine dihydrochloride. Veterinary Parasitology. 1998;75: 181–190. doi: 10.1016/s0304-4017(97)00197-0 9637219

[25] Prata LO, Rodrigues CR, Martins JM, Vasconcelos PC, Oliveira FMS, Ferreira AJ, et al. Original Research: ACE2 activator associated with physical exercise potentiates the reduction of pulmonary fibrosis. Experimental Biology and Medicine. 2017;242: 8–21. doi: 10.1177/1535370216665174 27550926 PMC5206984

[26] Collin AM, Lecocq M, Noel S, Detry B, Carlier FM, Aboubakar Nana F, et al. Lung immunoglobulin A immunity dysregulation in cystic fibrosis. EBioMedicine. 2020;60. doi: 10.1016/j.ebiom.2020.102974 32927272 PMC7495088

[27] Gazzinelli-guimarães AC, Gazzinelli-guimarães PH, Nogueira DS, Marcus F, Oliveira S, Barbosa FS, et al. IgG Induced by Vaccination With Ascaris suum Extracts Is Protective Against Infection. Frontiers in Immunology. 2018;9: 1–15. doi: 10.3389/fimmu.2018.00001 30473693 PMC6238660

[28] Ponce-Macotela M, Rodríguez-Caballero A, Peralta-Abarca GE, Martínez-Gordillo MN. A simplified method for hatching and isolating Toxocara canis larvae to facilitate excretory–secretory antigen collection in vitro. Veterinary Parasitology. 2011;175: 382–385. doi: 10.1016/j.vetpar.2010.10.030 21074327

[29] Silveira MR, Nunes KP, Cara DC, Souza DG, Correa A, Teixeira MM, et al. Infection with Strongyloides venezuelensis Induces Transient Airway Eosinophilic Inflammation, an Increase in Immunoglobulin E, and Hyperresponsiveness in Rats. Infection and Immunity. 2002;70: 6263–6272. doi: 10.1128/IAI.70.11.6263-6272.2002 12379705 PMC130296

[30] Strath M, Warren DJ, Sanderson CJ. Detection of eosinophils using an eosinophil peroxidase assay. Its use as an assay for eosinophil differentiation factors. Journal of Immunological Methods. 1985;83: 209–215. doi: 10.1016/0022-1759(85)90242-x 3840509

[31] Slawinska A, Dunislawska A, Plowiec A, Gonçalves J, Siwek M. TLR-mediated cytokine gene expression in chicken peripheral blood mononuclear cells as a measure to characterize immunobiotics. Genes. 2021;12. doi: 10.3390/genes12020195 33572768 PMC7912573

[32] El-Malky M, Maruyama H, Hirabayashi Y, Shimada S, Yoshida A, Amano T, et al. Intraepithelial infiltration of eosinophils and their contribution to the elimination of adult intestinal nematode, Strongyloides venezuelensis in mice. Parasitology International. 2003;52: 71–79. doi: 10.1016/s1383-5769(02)00086-7 12543149

[33] Bartemes KR, Cooper KM, Drain KL, Kita H. Secretory IgA induces antigen-independent eosinophil survival and cytokine production without inducing effector functions. Journal of Allergy and Clinical Immunology. 2005;116: 827–835. doi: 10.1016/j.jaci.2005.07.014 16210057

[34] Herr AB, White CL, Milburn C, Wu C, Bjorkman PJ. Bivalent binding of IgA1 to FcαRI suggests a mechanism for cytokine activation of IgA phagocytosis. Journal of Molecular Biology. 2003;327: 645–657. doi: 10.1016/s0022-2836(03)00149-9 12634059

[35] Pleass RJ, Lang ML, Kerr MA, Woof JM. IgA is a more potent inducer of NADPH oxidase activation and degranulation in blood eosinophils than IgE. Molecular Immunology. 2007;44: 1401–1408. doi: 10.1016/j.molimm.2006.05.002 16777227

[36] Rolot M, Dewals BG. Macrophage activation and functions during helminth infection: Recent advances from the laboratory mouse. Journal of Immunology Research. 2018. doi: 10.1155/2018/2790627 30057915 PMC6051086

[37] Inclan-Rico JM, Siracusa MC. First Responders: Innate Immunity to Helminths. Trends in Parasitology. 2018. doi: 10.1016/j.pt.2018.08.007 30177466 PMC6168350

[38] Coakley G, Harris NL. Interactions between macrophages and helminths. Parasite Immunology. 2020. doi: 10.1111/pim.12717 32249432

[39] Kawanishi H, Saltzman LE, Strober W. Mechanisms regulating iga class-specific immunoglobulin production in murine gut-associated lymphoid tissues: I. T cells derived from peyer’s patches that switch slgm b cells to slga B cells in vitro. Journal of Experimental Medicine. 1983;157. doi: 10.1084/jem.157.2.433 6185611 PMC2186919

[40] MacPherson AJ, McCoy KD, Johansen FE, Brandtzaeg P. The immune geography of IgA induction and function. Mucosal Immunology. 2008. doi: 10.1038/mi.2007.6 19079156

[41] FitzPatrick RD, Kennedy MHE, Lawrence KM, Gauthier CM, Moeller BE, Robinson AN, et al. Littermate-controlled experiments reveal eosinophils are not essential for maintaining steady-state IgA and demonstrate the influence of rearing conditions on antibody phenotypes in eosinophil-deficient mice. Frontiers in Immunology. 2020;11: 1–10. doi: 10.3389/fimmu.2020.00001 33178185 PMC7593696

[42] Beller A, Kruglov A, Durek P, Goetze V, Werner K, Heinz GA, et al. Specific microbiota enhances intestinal IgA levels by inducing TGF-β in T follicular helper cells of Peyer’s patches in mice. European Journal of Immunology. 2020;50: 783–794. doi: 10.1002/eji.201948474 32065660

[43] Forman R, Bramhall M, Logunova L, Svensson-Frej M, Cruickshank SM, Else KJ. Eosinophils may play regionally disparate roles in influencing IgA+ plasma cell numbers during large and small intestinal inflammation. BMC Immunology. 2016;17: 12. doi: 10.1186/s12865-016-0153-0 27245920 PMC4886441

[44] Masure D, Vlaminck J, Wang T, Chiers K, van den Broeck W, Vercruysse J, et al. A Role for Eosinophils in the Intestinal Immunity against Infective Ascaris suum Larvae. PLoS Neglected Tropical Diseases. 2013;7: 1–7. doi: 10.1371/journal.pntd.0002138 23556022 PMC3605247

[45] Masure D, Wang T, Vlaminck J, Claerhoudt S, Chiers K, van den Broeck W, et al. The Intestinal Expulsion of the Roundworm Ascaris suum Is Associated with Eosinophils, Intra-Epithelial T Cells and Decreased Intestinal Transit Time. PLoS Neglected Tropical Diseases. 2013;7: 1–9. doi: 10.1371/journal.pntd.0002588 24340121 PMC3854935

[46] Balic A, Cunningham CP, Meeusen ENT. Eosinophil interactions with Haemonchus contortus larvae in the ovine gastrointestinal tract. Parasite Immunology. 2006;28: 107–115. doi: 10.1111/j.1365-3024.2006.00816.x 16441509

[47] Goh YPS, Henderson NC, Heredia JE, Eagle AR, Odegaard JI, Lehwald N, et al. Eosinophils secrete IL-4 to facilitate liver regeneration. Proceedings of the National Academy of Sciences of the United States of America. 2013;110: 9914–9919. doi: 10.1073/pnas.1304046110 23716700 PMC3683773

[48] Isobe Y, Kato T, Arita M. Emerging roles of eosinophils and eosinophil-derived lipid mediators in the resolution of inflammation. Frontiers in Immunology. 2012;3: 1–6. doi: 10.3389/fimmu.2012.00001 22973272 PMC3428698

[49] Reyes JL, Espinoza-Jiménez AF, González MI, Verdin L, Terrazas LI. Taenia crassiceps infection abrogates experimental autoimmune encephalomyelitis. Cellular Immunology. 2011;267. doi: 10.1016/j.cellimm.2010.11.006 21185554

[50] Zhang M, Gao Y, Du X, Zhang D, Ji M, Wu G. Toll-like receptor (TLR) 2 and TLR4 deficiencies exert differential in vivo effects against Schistosoma japonicum. Parasite Immunology. 2011;33. doi: 10.1111/j.1365-3024.2010.01265.x 21392041

[51] van der Kleij D, Latz E, Brouwers JFHM, Kruize YCM, Schmitz M, Kurt-Jones EA, et al. A novel host-parasite lipid cross-talk. Schistosomal lyso-phosphatidylserine activates toll-like receptor 2 and affects immune polarization. Journal of Biological Chemistry. 2002;277. doi: 10.1074/jbc.M206941200 12359728

[52] Kerepesi LA, Leon O, Lustigman S, Abraham D. Protective immunity to the larval stages of Onchocerca volvulus is dependent on toll-like receptor 4. Infection and Immunity. 2005;73. doi: 10.1128/IAI.73.12.8291-8297.2005 16299326 PMC1307100

[53] Kerepesi LA, Hess JA, Leon O, Nolan TJ, Schad GA, Abraham D. Toll-like receptor 4 (TLR4) is required for protective immunity to larval Strongyloides stercoralis in mice. Microbes and Infection. 2007;9. doi: 10.1016/j.micinf.2006.10.003 17196865

[54] Lanzavecchia A, Sallusto F. Toll-like receptors and innate immunity in B-cell activation and antibody responses. Current Opinion in Immunology. 2007;19: 268–274. doi: 10.1016/j.coi.2007.04.002 17433875

[55] Onguru D, Liang YM, Griffith Q, Nikolajczyk B, Mwinzi P, Ganley-Leal L. Short report: Human schistosomiasis is associated with endotoxemia and toll-like receptor 2- and 4-bearing B cells. American Journal of Tropical Medicine and Hygiene. 2011;84: 321–324. doi: 10.4269/ajtmh.2011.10–0397 PMC3029191 21292908

[56] Kopf M, Brombacher F, Hodgkin PD, Ramsay AJ, Milbourne EA, Dai WJ, et al. IL-5-Deficient mice have a developmental defect in CD5+ B-1 cells and lack eosinophilia but have normal antibody and cytotoxic T cell responses. Immunity. 1996;4. doi: 10.1016/s1074-7613(00)80294-0 8574848

[57] Finkelman FD, Holmes J, Katona IM, Urban JF, Beckmann MP, Park LS, et al. Lymphokine control of in vivo immunoglobulin isotype selection. Annual Review of Immunology. 1990. doi: 10.1146/annurev.iy.08.040190.001511 1693082

[58] Schoenbeck S, Mckenzie DT, Kagnoff MF. Interleukin 5 is a differentiation factor for IgA B cells. European Journal of Immunology. 1989;19. doi: 10.1002/eji.1830190602 2787753

[59] Deslyper G, Colgan TJ, Cooper AJR, Holland C v., Carolan JC. A Proteomic Investigation of Hepatic Resistance to Ascaris in a Murine Model. PLoS Neglected Tropical Diseases. 2016;10: 1–26. doi: 10.1371/journal.pntd.0004837 27490109 PMC4974003

[60] Zuo L, Lucas K, Fortuna CA, Chuang C, Best TM. Molecular Regulation of Toll-like Receptors in Asthma and COPD. Frontiers in Physiology. 2015;6: 1–10. doi: 10.3389/fphys.2015.00001 26617525 PMC4637409

[61] Pham D le Yoon MG, Ban GY Kim SH, Kim MA Ye YM, et al. Serum S100A8 and S100A9 enhance innate immune responses in the pathogenesis of baker’s asthma. International Archives of Allergy and Immunology. 2015;168: 138–146. doi: 10.1159/000441678 26745257

[62] Ludwig-Portugall I, Layland LE. TLRs, Treg, and B Cells, an Interplay of Regulation during Helminth Infection. Frontiers in Immunology. 2012;3: 1–7. doi: 10.3389/fimmu.2012.00001 22566894 PMC3342019

[63] Ruprecht CR, Lanzavecchia A. Toll-like receptor stimulation as a third signal required for activation of human naive B cells. European Journal of Immunology. 2006;36. doi: 10.1002/eji.200535744 16541472

[64] Alugupalli KR, Abraham D. B Cell Multitasking Is Required to Control Nematode Infection. Immunity. 2009;30: 317–319. doi: 10.1016/j.immuni.2009.02.004 19303384

[65] Alugupalli KR, Leong JM, Woodland RT, Muramatsu M, Honjo T, Gerstein RM. B1b lymphocytes confer T cell-independent long-lasting immunity. Immunity. 2004;21: 379–390. doi: 10.1016/j.immuni.2004.06.019 15357949

[66] Alugupalli KR. A distinct role for B1b lymphocytes in T cell-independent immunity. Current Topics in Microbiology and Immunology. 2008;319: 105–130. doi: 10.1007/978-3-540-73900-5_5 18080416

[67] Gunti S, Messer RJ, Xu C, Yan M, Jr WGC, Peterson KE, et al. Stimulation of Toll-Like Receptors profoundly influences the titer of polyreactive antibodies in the circulation. Nature Publishing Group. 2015; 1–8. doi: 10.1038/srep15066 26463758 PMC4604466

[68] Peterson LW, Artis D. Intestinal epithelial cells: Regulators of barrier function and immune homeostasis. Nature Reviews Immunology. 2014;14: 141–153. doi: 10.1038/nri3608 24566914

[69] Brandtzaeg P. Secretory IgA: Designed for anti-microbial defense. Frontiers in Immunology. 2013;4: 1–17. doi: 10.3389/fimmu.2013.00001 23964273 PMC3734371

[70] de Jesus LCL, Drumond MM, Aburjaile FF, Sousa T de J, Coelho-Rocha ND, Profeta R, et al. Probiogenomics of lactobacillus delbrueckii subsp. Lactis cidca 133: In silico, in vitro, and in vivo approaches. Microorganisms. 2021;9. doi: 10.3390/microorganisms9040829 33919849 PMC8070793

[71] Maldonado Galdeano C, Perdigón G. The probiotic bacterium Lactobacillus casei induces activation of the gut mucosal immune system through innate immunity. Clinical and Vaccine Immunology. 2006;13. doi: 10.1128/CVI.13.2.219-226.2006 16467329 PMC1391937

[72] Rajput IR, Ying H, Yajing S, Arain MA, Weifen L, Ping L, et al. Correction: Saccharomyces boulardii and Bacillus subtilis B10 modulate TLRs and cytokines expression patterns in jejunum and ileum of broilers (PLoS ONE (2017) 12:6 (e0173917) PLoS ONE. 2017. doi: 10.1371/journal.pone.0180752 28319123 PMC5358784

[73] Sakai F, Hosoya T, Ono-Ohmachi A, Ukibe K, Ogawa A, Moriya T, et al. Lactobacillus gasseri SBT2055 induces TGF-β expression in dendritic cells and activates TLR2 signal to produce IgA in the small intestine. PLoS ONE. 2014;9. doi: 10.1371/journal.pone.0105370 25144744 PMC4140756

[74] Sugawara R, Lee EJ, Jang MS, Jeun EJ, Hong CP, Kim JH, et al. Small intestinal eosinophils regulate Th17 cells by producing IL-1 receptor antagonist. Journal of Experimental Medicine. 2016;213. doi: 10.1084/jem.20141388 26951334 PMC4821642

[75] Strandmark J, Steinfelder S, Berek C, Kühl AA, Rausch S, Hartmann S. Eosinophils are required to suppress Th2 responses in Peyer’s patches during intestinal infection by nematodes. Mucosal Immunology. 2017;10. doi: 10.1038/mi.2016.93 27805618

[76] Olbrich CL, Bivas-Benita M, Xenakis JJ, Maldonado S, Cornwell E, Fink J, et al. Remote allergen exposure elicits eosinophil infiltration into allergen nonexposed mucosal organs and primes for allergic inflammation. Mucosal Immunology. 2020;13. doi: 10.1038/s41385-020-0310-x 32518365 PMC7442625

[77] Fransen F, Zagato E, Mazzini E, Fosso B, Manzari C, el Aidy S, et al. BALB/c and C57BL/6 Mice Differ in Polyreactive IgA Abundance, which Impacts the Generation of Antigen-Specific IgA and Microbiota Diversity. Immunity. 2015;43. doi: 10.1016/j.immuni.2015.08.011 26362264

