# Supplementary figures and images for: Eosinophils mediate SIgA production triggered by TLR2 and TLR4 to control Ascaris suum infection in mice
Source: PLoS Pathog. 2021 Nov 16;17(11):e1010067. doi: 10.1371/journal.ppat.1010067 (PMC8631680; doi:10.1371/journal.ppat.1010067)

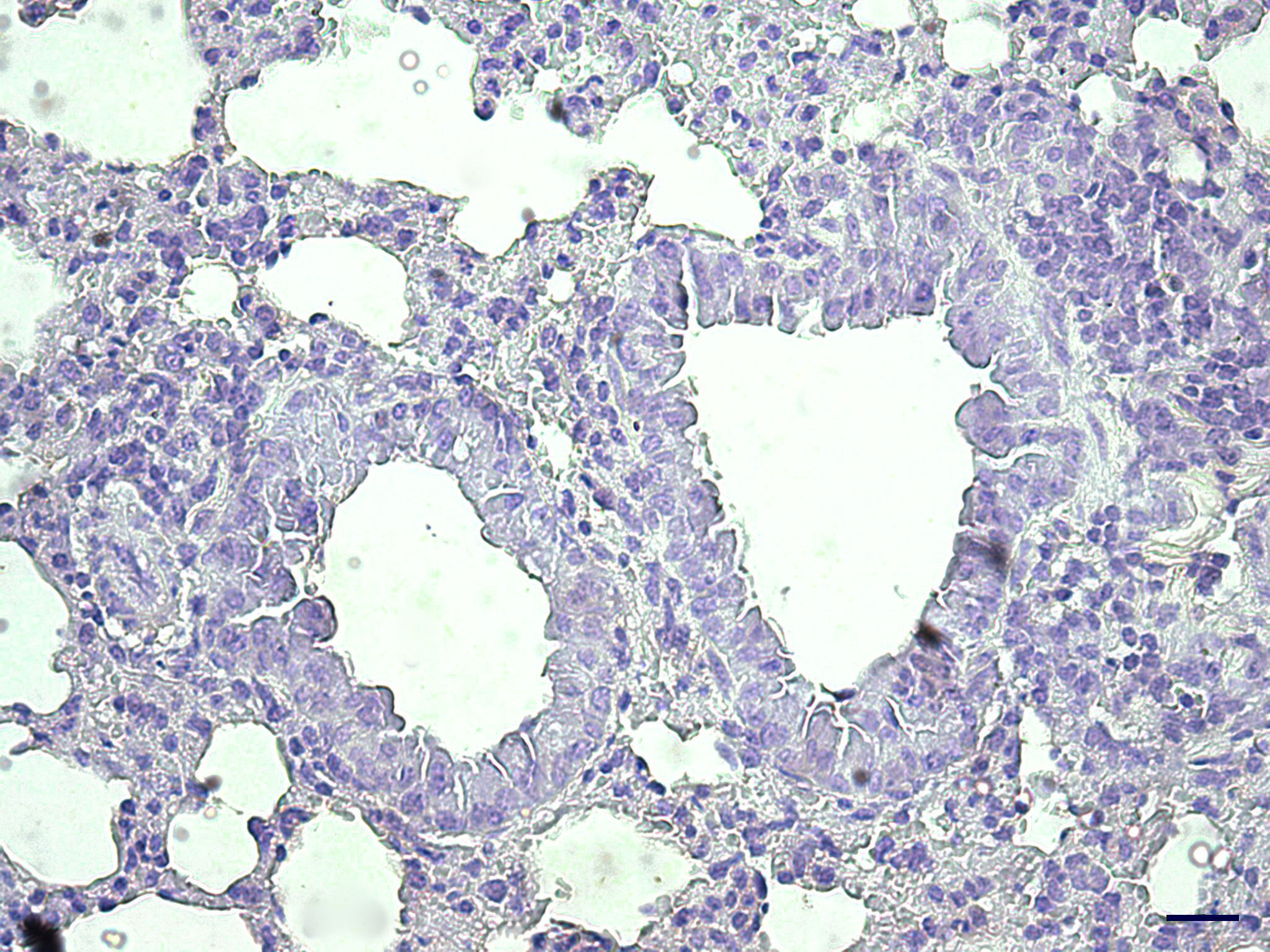

Supplement: S1 Fig — Histological lung sections of re-infected WT BALB/c mice. (TIF) [file ppat.1010067.s001.tif]

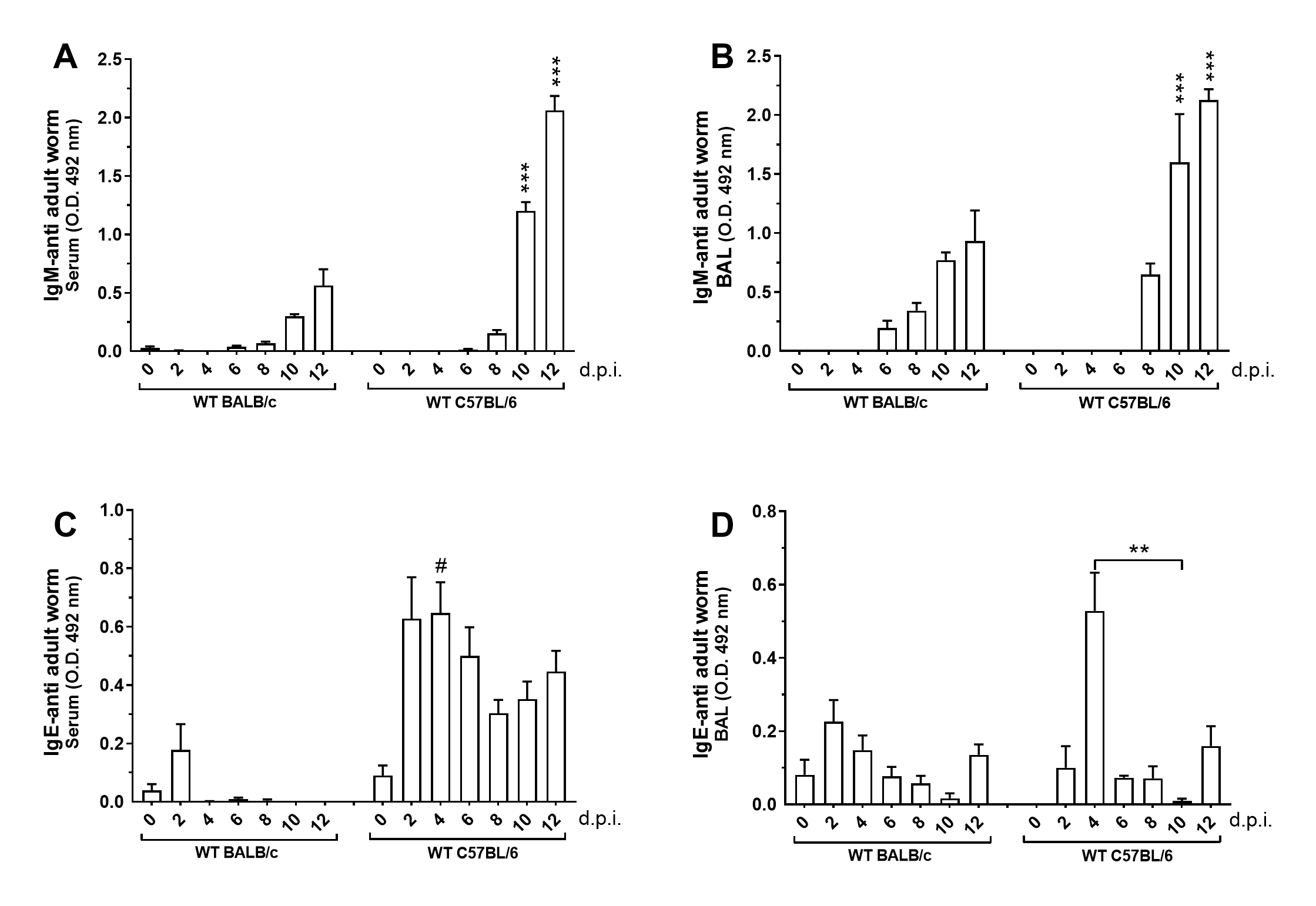

Supplement: S2 Fig — Data are represented as mean ± SEM. The Kruskal-Wallis test followed by Dunn´s multiple comparisons test was used to evaluate differences between groups (A-D). Significant differences (p < 0.05) are represented in the graph by symbols. * represents differences between the evaluated time and the previous times in the same strains of mice; # represents the differences between different strains at the same time of infection. [1 symbol = p < 0.05], [2 symbols = p < 0.01], and [3 symbols = p < 0.001]. (TIF) [file ppat.1010067.s002.tif]

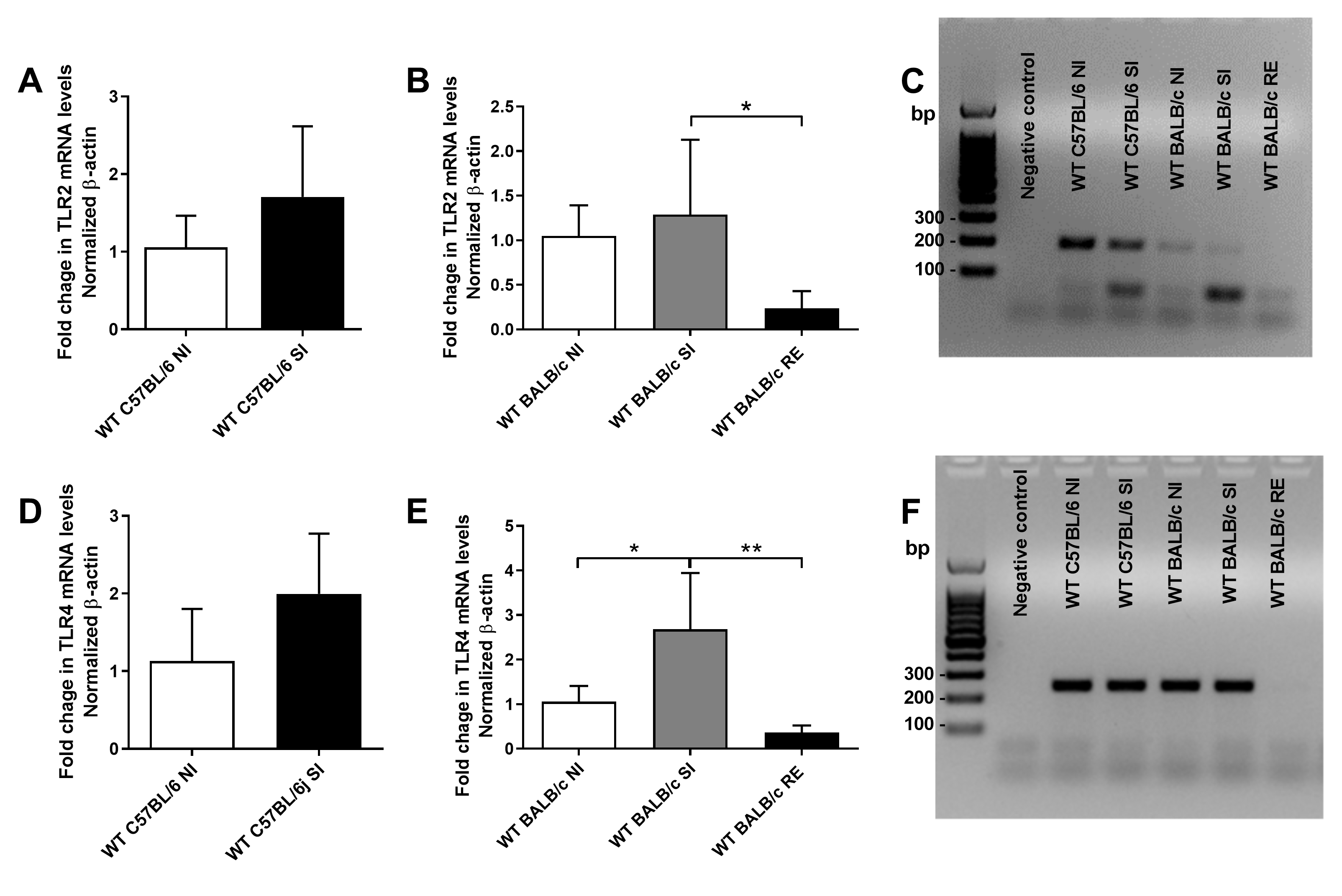

Supplement: S3 Fig — RT-qPCR data were obtained using the cDNA of non-infected (NI) and single-infected (SI) BALB/c and C57BL/6 mice and re-infected (RE) BALB/c mice (n = 5 / group). The relative expression of TLR-2 and TLR-4 mRNA was normalized to the reference gene β-actin and the fold change was calculated using the 2(-ΔΔCt) method. The fold change was expressed as mean ± standard deviation for all groups. The Mann-Whitney test was used to evaluate the differences between the groups (A and D). One-way ANOVA followed by Tukey´s multiple comparisons test was used to evaluate the differences between groups (B and E). Statistical differences are represented by symbols in the graphs, where * represents p < 0.05, ** represents p < 0.01. All experiments were performed on the 8th dpi. RT-qPCR products were analyzed using 2% agarose gel electrophoresis. The 191 bp and 249 bp bands amplicons correspond to the amplified fragments of the TLR-2 (C) and TLR-4 (F) cDNA, respectively. bp: base pairs. (TIF) [file ppat.1010067.s003.tif]

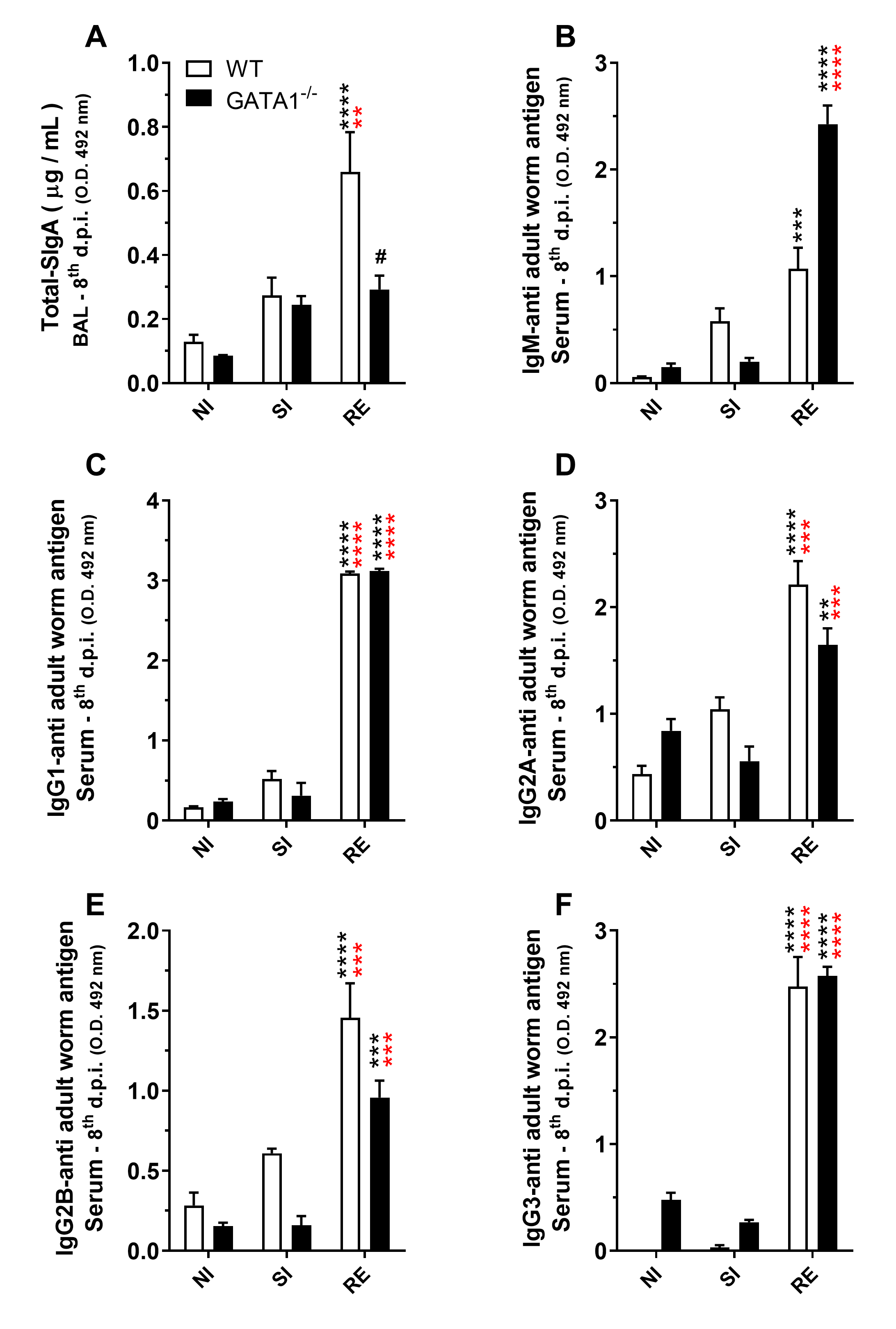

Supplement: S4 Fig — P values are represented by symbols in the graphs wherein * represents differences between the non-infected groups of the same strain, * represents differences between the single-infected groups of the same strain, # represents differences between groups from different strains that received the same treatment. One-way ANOVA followed by Tukey´s multiple comparisons test was used to evaluate differences between groups (A-F). [1 symbol = p < 0.05], [2 symbols = p < 0.01], [3 symbols = p < 0.001], and [4 symbols = p < 0.0001]. (TIF) [file ppat.1010067.s004.tif]
