# Supplementary material for: Eosinophils mediate SIgA production triggered by TLR2 and TLR4 to control Ascaris suum infection in mice
Source: PLoS Pathog. 2021 Nov 16;17(11):e1010067. doi: 10.1371/journal.ppat.1010067 (PMC8631680; doi:10.1371/journal.ppat.1010067)
Supplement: S1 Table — Significant p-values (p < 0.05) are indicated in bold. One-way ANOVA followed by Tukey´s multiple comparisons test was used to evaluate the differences between times. (DOCX) [file ppat.1010067.s005.docx]

| **Times (dpi)** | | **Total Leukocytes** | | | | | | |  |
| --- | --- | --- | --- | --- | --- | --- | --- | --- | --- |
|  |  | 0 | 2 | 4 | 6 | 8 | 10 | 12 |  |
| **Total Leukocytes** | 0 |  | > 0.9999 | > 0.9999 | 0.9995 | **< 0.0001** | **< 0.0001** | **< 0.0001** |  |
|  | 2 | > 0.9999 |  | > 0.9999 | 0.9996 | **< 0.0001** | **< 0.0001** | **< 0.0001** |  |
|  | 4 | > 0.9999 | > 0.9999 |  | > 0.9999 | **< 0.0001** | **< 0.0001** | **< 0.0001** |  |
|  | 6 | 0.9995 | 0.9996 | > 0.9999 |  | **< 0.0001** | **< 0.0001** | **< 0.0001** |  |
|  | 8 | **< 0.0001** | **< 0.0001** | **< 0.0001** | **< 0.0001** |  | 0.8809 | **0.0251** |  |
|  | 10 | **< 0.0001** | **< 0.0001** | **< 0.0001** | **< 0.0001** | 0.8809 |  | **0.4239** |  |
|  | 12 | **< 0.0001** | **< 0.0001** | **< 0.0001** | **< 0.0001** | **0.0251** | **0.4239** |  |  |
|  |  |  |  |  |  |  |  |  |  |
| **Times (dpi)** | | **Macrophages** | | | | | | |  |
|  |  | 0 | 2 | 4 | 6 | 8 | 10 | 12 |  |
| **Macrophages** | 0 |  | > 0.9999 | 0.9997 | 0.9443 | **< 0.0001** | **< 0.0001** | **< 0.0001** |  |
|  | 2 | > 0.9999 |  | 0.9997 | 0.958 | **< 0.0001** | **< 0.0001** | **< 0.0001** |  |
|  | 4 | 0.9997 | 0.9997 |  | 0.9974 | **0.0002** | **< 0.0001** | **< 0.0001** |  |
|  | 6 | 0.9443 | 0.958 | 0.9974 |  | **0.001** | **< 0.0001** | **< 0.0001** |  |
|  | 8 | **< 0.0001** | **< 0.0001** | **0.0002** | **0.001** |  | **0.0089** | **0.0251** |  |
|  | 10 | **< 0.0001** | **< 0.0001** | **< 0.0001** | **< 0.0001** | **0.0089** |  | 0.6179 |  |
|  | 12 | **< 0.0001** | **< 0.0001** | **< 0.0001** | **< 0.0001** | **0.0251** | 0.6179 |  |  |
|  |  |  |  |  |  |  |  |  |  |
| **Times** | | **Lymphocytes** | | | | | | |  |
|  |  | 0 | 2 | 4 | 6 | 8 | 10 | 12 |  |
| **Lymphocytes** | 0 |  | > 0.9999 | > 0.9999 | 0.9737 | **0.0036** | **0.0017** | **< 0.0001** |  |
|  | 2 | > 0.9999 |  | > 0.9999 | 0.9831 | **0.0101** | **0.0053** | **< 0.0001** |  |
|  | 4 | > 0.9999 | > 0.9999 |  | 0.9853 | **0.0092** | **0.0048** | **< 0.0001** |  |
|  | 6 | 0.9737 | 0.9831 | 0.9853 |  | **0.083** | **0.0495** | **< 0.0001** |  |
|  | 8 | **0.0036** | **0.0101** | **0.0092** | **0.083** |  | **> 0.9999** | **< 0.0001** |  |
|  | 10 | **0.0017** | **0.0053** | **0.0048** | **0.0495** | > 0.9999 |  | **< 0.0001** |  |
|  | 12 | **< 0.0001** | **< 0.0001** | **< 0.0001** | **< 0.0001** | **< 0.0001** | **< 0.0001** |  |  |
|  |  |  |  |  |  |  |  |  |  |
| **Times** | | **Neutrophils** | | | | | | |  |
|  |  | 0 | 2 | 4 | 6 | 8 | 10 | 12 |  |
| **Neutrophils** | 0 |  | > 0.9999 | > 0.9999 | 0.694 | **< 0.0001** | **< 0.0001** | 0.1965 |  |
|  | 2 | > 0.9999 |  | > 0.9999 | 0.7595 | **< 0.0001** | **< 0.0001** | 0.2677 |  |
|  | 4 | > 0.9999 | > 0.9999 |  | 0.7707 | **< 0.0001** | **< 0.0001** | 0.2773 |  |
|  | 6 | 0.694 | 0.7595 | 0.7707 |  | **< 0.0001** | **< 0.0001** | 0.9814 |  |
|  | 8 | **< 0.0001** | **< 0.0001** | **< 0.0001** | **< 0.0001** |  | **< 0.0001** | **< 0.0001** |  |
|  | 10 | **< 0.0001** | **< 0.0001** | **< 0.0001** | **< 0.0001** | **< 0.0001** |  | **< 0.0001** |  |
|  | 12 | 0.1965 | 0.2677 | 0.2773 | 0.9814 | **< 0.0001** | **< 0.0001** |  |  |
|  |  |  |  |  |  |  |  |  |  |
| **Times** | | **Eosinophils** | | | | | | |  |
|  |  | 0 | 2 | 4 | 6 | 8 | 10 | 12 |  |
| **Eosinophils** | 0 |  | > 0.9999 | > 0.9999 | > 0.9999 | 0.8962 | **< 0.0001** | **< 0.0001** |  |
|  | 2 | > 0.9999 |  | > 0.9999 | > 0.9999 | 0.9227 | **< 0.0001** | **< 0.0001** |  |
|  | 4 | > 0.9999 | > 0.9999 |  | > 0.9999 | 0.9291 | **< 0.0001** | **< 0.0001** |  |
|  | 6 | > 0.9999 | > 0.9999 | > 0.9999 |  | 0.9665 | 0.0002 | **< 0.0001** |  |
|  | 8 | 0.8962 | 0.9227 | 0.9291 | 0.9665 |  | 0.0058 | **< 0.0001** |  |
|  | 10 | **< 0.0001** | **< 0.0001** | **< 0.0001** | **0.0002** | **0.0058** |  | **0.0002** |  |
|  | 12 | **< 0.0001** | **<0.0001** | **< 0.0001** | **<0.0001** | **< 0.0001** | **0.0002** |  |  |
|  |  |  |  |  |  |  |  |  |  |
| **S1 Table:** Statistical differences in the total number of leukocytes and their cell subpopulations in bronchoalveolar lavage fluid at different times of infection. Significant p values are represented in bold (p < 0.05). One-Way ANOVA followed by Tukey´s multiple comparisons test was used to evaluate differences between times. | | | | | | | | |  |
|  |  |  |  |  |  |  |  |  |  |
|  |  |  |  |  |  |  |  |  |  |
