# Supplementary material for: Eosinophils mediate SIgA production triggered by TLR2 and TLR4 to control Ascaris suum infection in mice
Source: PLoS Pathog. 2021 Nov 16;17(11):e1010067. doi: 10.1371/journal.ppat.1010067 (PMC8631680; doi:10.1371/journal.ppat.1010067)
Supplement: S2 Table — Groups that presented higher levels of SIgA are shown on the left. Statistical differences are indicated by p-values and symbols in bold. Two-way ANOVA followed by Sidak’s multiple comparisons test was used to evaluate differences between groups. (DOCX) [file ppat.1010067.s006.docx]

| Sidak's multiple comparisons test | Significant? | Summary | P value |
| --- | --- | --- | --- |
|  |  |  |  |
| BALB/c NI: NCDO vs. BALB/c NI: Placebo | **Yes** | ******* | **0.0002** |
| GATA1^-/-^ NI: Placebo vs. BALB/c NI: Placebo | **Yes** | ******* | **0.0004** |
| BALB/c NI: Placebo vs. GATA1^-/-^ NI: NCDO | Yes | **** | <0.0001 |
| BALB/c SI: Placebo vs. BALB/c NI: Placebo | **Yes** | ******** | **<0.0001** |
| BALB/c NI: Placebo vs. BALB/c SI: NCDO | Yes | **** | <0.0001 |
| BALB/c NI: Placebo vs. GATA1^-/-^ SI: Placebo | No | ns | 0.9995 |
| BALB/c NI: Placebo vs. GATA1^-/-^ SI: NCDO | Yes | **** | <0.0001 |
| BALB/c NI: NCDO vs. GATA1^-/-^ NI: Placebo | No | ns | >0.9999 |
| BALB/c NI: NCDO vs. GATA1^-/-^ NI: NCDO | No | ns | 0.8137 |
| BALB/c NI: NCDO vs. BALB/c SI: Placebo | No | ns | 0.9985 |
| BALB/c NI: NCDO vs. BALB/c SI: NCDO | No | ns | 0.9954 |
| BALB/c NI: NCDO vs. GATA1^-/-^ SI: Placebo | Yes | * | 0.0472 |
| BALB/c NI: NCDO vs. GATA1^-/-^ SI: NCDO | No | ns | 0.8353 |
| GATA1^-/-^ NI: Placebo vs. GATA1^-/-^ NI: NCDO | No | ns | 0.9994 |
| GATA1^-/-^ NI: Placebo vs. BALB/c SI: Placebo | No | ns | >0.9999 |
| GATA1^-/-^ NI: Placebo vs. BALB/c SI: NCDO | No | ns | >0.9999 |
| GATA1^-/-^ NI: Placebo vs. GATA1^-/-^ SI: Placebo | **Yes** | ***** | **0.0370** |
| GATA1^-/-^ NI: Placebo vs. GATA1^-/-^ SI: NCDO | No | ns | 0.9996 |
| GATA1^-/-^ NI: NCDO vs. BALB/c SI: Placebo | No | ns | >0.9999 |
| GATA1^-/-^ NI: NCDO vs. BALB/c SI: NCDO | No | ns | >0.9999 |
| GATA1^-/-^ NI: NCDO vs. GATA1^-/-^ SI: Placebo | Yes | *** | 0.0006 |
| GATA1^-/-^ NI: NCDO vs. GATA1^-/-^ SI: NCDO | No | ns | >0.9999 |
| BALB/c SI: Placebo vs. BALB/c SI: NCDO | No | ns | >0.9999 |
| BALB/c SI: Placebo vs. GATA1^-/-^ SI: Placebo | **Yes** | ****** | **0.0016** |
| BALB/c SI: Placebo vs. GATA1^-/-^ SI: NCDO | No | ns | >0.9999 |
| BALB/c SI: NCDO vs. GATA1^-/-^ SI: Placebo | Yes | ** | 0.0015 |
| BALB/c SI: NCDO vs. GATA1^-/-^ SI: NCDO | No | ns | >0.9999 |
| GATA1^-/-^ SI: NCDO vs. GATA1^-/-^ SI: Placebo | **Yes** | ******* | **0.0006** |

**S2 Table:** Statistical differences in Total SIgA in the BAL 8 d.p.i. with *Ascaris suum*, and after 22 days of treatment with 1x10^9 CFU/mL^ of *Lactococcus lactis* (NCDO (2118) or Placebo (PBS). Groups that presented higher levels of SIgA are cited on the left. Statistical differences are represented by p values and symbols in bold in the table. Two-Way ANOVA followed by Sidak's multiple comparisons test was used to evaluate differences between groups.
