# Supplementary material for: Eosinophils mediate SIgA production triggered by TLR2 and TLR4 to control Ascaris suum infection in mice
Source: PLoS Pathog. 2021 Nov 16;17(11):e1010067. doi: 10.1371/journal.ppat.1010067 (PMC8631680; doi:10.1371/journal.ppat.1010067)
Supplement: S3 Table — Two-way ANOVA followed by Sidak’s multiple comparisons test was used to evaluate differences between groups. (DOCX) [file ppat.1010067.s007.docx]

| Sidak's multiple comparisons test | Significant? | Summary | P value |
| --- | --- | --- | --- |
|  |  |  |  |
| BALB/c NI: Placebo vs. BALB/c NI:NCDO | No | ns | 0.9269 |
| BALB/c NI: Placebo vs. GATA1^-/-^ NI: Placebo | No | ns | 0.9938 |
| BALB/c NI: Placebo vs. GATA1^-/-^ NI:NCDO | No | ns | 0.8428 |
| BALB/c NI: Placebo vs. BALB/c SI: Placebo | No | ns | >0.9999 |
| BALB/c NI: Placebo vs. BALB/c SI:NCDO | No | ns | >0.9999 |
| BALB/c NI: Placebo vs. GATA1^-/-^ SI: Placebo | No | ns | 0.8441 |
| BALB/c NI: Placebo vs. GATA1^-/-^ SI:NCDO | No | ns | 0.3140 |
| BALB/c NI: NCDO vs. GATA1^-/-^ NI: Placebo | No | ns | >0.9999 |
| BALB/c NI: NCDO vs. GATA1^-/-^ NI:NCDO | No | ns | >0.9999 |
| BALB/c NI: NCDO vs. BALB/c SI: Placebo | No | ns | 0.2474 |
| BALB/c NI: NCDO vs. BALB/c SI:NCDO | No | ns | 0.7048 |
| BALB/c NI: NCDO vs. GATA1^-/-^ SI: Placebo | No | ns | >0.9999 |
| BALB/c NI: NCDO vs. GATA1^-/-^ SI:NCDO | No | ns | 0.9996 |
| GATA1^-/-^ NI: Placebo vs. GATA1^-/-^ NI:NCDO | No | ns | >0.9999 |
| GATA1^-/-^ NI: Placebo vs. BALB/c SI: Placebo | No | ns | 0.6359 |
| GATA1^-/-^ NI: Placebo vs. BALB/c SI:NCDO | No | ns | 0.9419 |
| GATA1^-/-^ NI: Placebo vs. GATA1^-/-^ SI: Placebo | No | ns | >0.9999 |
| GATA1^-/-^ NI: Placebo vs. GATA1^-/-^ SI:NCDO | No | ns | >0.9999 |
| GATA1^-/-^ NI: NCDO vs. BALB/c SI: Placebo | No | ns | 0.2573 |
| GATA1^-/-^ NI: NCDO vs. BALB/c SI:NCDO | No | ns | 0.6200 |
| GATA1^-/-^ NI: NCDO vs. GATA1^-/-^ SI: Placebo | No | ns | >0.9999 |
| GATA1^-/-^ NI: NCDO vs. GATA1^-/-^ SI:NCDO | No | ns | >0.9999 |
| BALB/c SI: Placebo vs. BALB/c SI:NCDO | No | ns | >0.9999 |
| BALB/c SI: Placebo vs. GATA1^-/-^ SI: Placebo | No | ns | 0.2257 |
| BALB/c SI: Placebo vs. GATA1^-/-^ SI:NCDO | Yes | * | 0.0445 |
| BALB/c SI: NCDO vs. GATA1^-/-^ SI: Placebo | No | ns | 0.6043 |
| BALB/c SI: NCDO vs. GATA1^-/-^ SI:NCDO | No | ns | 0.1641 |
| GATA1^-/-^ SI: Placebo vs. GATA1^-/-^ SI:NCDO | No | ns | >0.9999 |

**S3 Table:** Statistical differences in Total SIgA in the Intestine lavage 8 d.p.i. with *Ascaris suum*, and after 22 days of treatment with 1x10^9^ of *Lactococcus lactis* (NCDO (2118)) or Placebo. Two-Way ANOVA followed by Sidak's multiple comparisons test was used to evaluate differences between groups.
